# 12-Tungstophospho­ric acid–1,1′-methyl­enebis(imidazolidine-2,4-dione)–imidazolidine-2,4-dione–water (2/1/4/21.5)

**DOI:** 10.1107/S160053681002310X

**Published:** 2010-06-23

**Authors:** Mohammad Hasan Alizadeh, Irandokht Mohammadi Zonoz

**Affiliations:** a Department of Chemistry, Ferdowsi University of Mashhad, Mashhad, 91779, Iran

## Abstract

In the asymmetric unit of the title organic–inorganic hybrid material, 2{H_3_[PW_12_O_40_]}·4C_3_H_4_N_2_O_2_·C_7_H_8_N_4_O_4_·21.5H_2_O, there are four crystallographically independent hydantoin mol­ecules, one dimerized hydantoin mol­ecule, *viz.* 1,1′-methyl­enebis(imidazolidine-2,4-dione, two independent H_3_PW_12_O_40_ mol­ecules and 21.5 solvent water mol­ecules. Nine of the solvent water mol­ecules were refined as 0.5 occupancy. The tungstophosphoric acid moieties show characteristic features with respect to the bond lengths and angles.

## Related literature

For the medical applications of some hydantoin derivatives as anti­epileptic drugs, see: Micali *et al.* (1999 ▶. For the applications of polyoxometalates, see: Yanagie *et al.* (2006 ▶). For background to organic–inorganic hybrid materials, see: Guangzhe *et al.* (2008 ▶). The heteropoly acid molecule (Keggin structure) consists of a central PO_4_ tetra­hedron surrounded by twelve WO_6_ octa­hedra, see: Keggin (1933 ▶). For a related Keggin structure, see: Lebeden *et al.* (1969 ▶). 
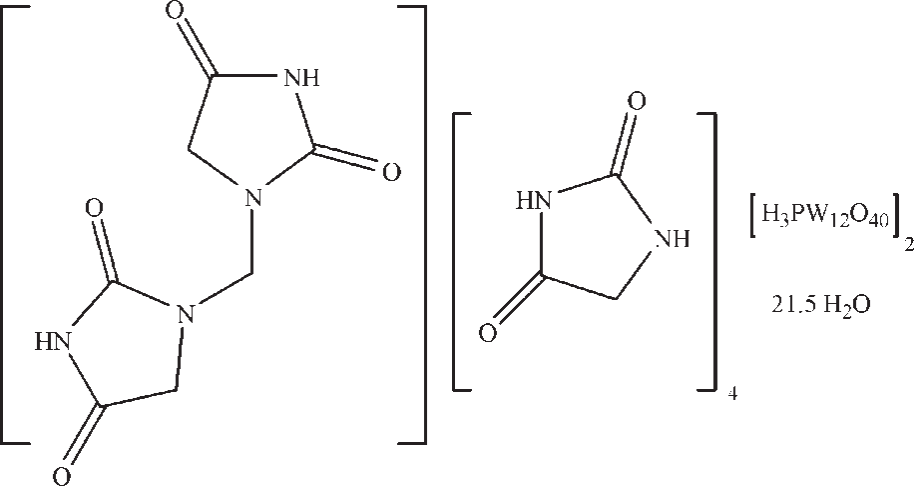


## Experimental

### 

#### Crystal data

2{H_3_[PW_12_O_40_]}·4C_3_H_4_N_2_O_2_·C_7_H_8_N_4_O_4_·21.5H_2_O
*M* *_r_* = 6760.23
Triclinic, 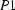

*a* = 12.7271 (6) Å
*b* = 13.3286 (7) Å
*c* = 18.9177 (9) Å
α = 71.328 (1)°
β = 81.316 (1)°
γ = 64.751 (1)°
*V* = 2749.3 (2) Å^3^
*Z* = 1
Mo *K*α radiation
μ = 25.15 mm^−1^
*T* = 100 K
0.31 × 0.17 × 0.08 mm

#### Data collection

Bruker SMART APEXII CCD diffractometer
Absorption correction: integration (*SADABS*; Bruker, 2005 ▶) *T*_min_ = 0.031, *T*_max_ = 0.215
51543 measured reflections
25728 independent reflections
22772 reflections with *I* > 2σ(*I*)
*R*_int_ = 0.048

#### Refinement

*R*[*F* ^2^ > 2σ(*F* ^2^)] = 0.043
*wR*(*F* ^2^) = 0.102
*S* = 1.01
25728 reflections
1577 parameters
45 restraints
H-atom parameters constrained
Δρ_max_ = 2.44 e Å^−3^
Δρ_min_ = −1.32 e Å^−3^
Absolute structure: Flack (1983 ▶), 12649 Friedel pairs
Flack parameter: 0.492 (11)

### 

Data collection: *APEX2* (Bruker, 2005 ▶); cell refinement: *SAINT* (Bruker, 2005 ▶); data reduction: *SAINT*; program(s) used to solve structure: *SHELXTL* (Sheldrick, 2008 ▶); program(s) used to refine structure: *SHELXTL*; molecular graphics: *SHELXTL*; software used to prepare material for publication: *SHELXTL*.

## Supplementary Material

Crystal structure: contains datablocks I, global. DOI: 10.1107/S160053681002310X/lh5037sup1.cif

Structure factors: contains datablocks I. DOI: 10.1107/S160053681002310X/lh5037Isup2.hkl

Additional supplementary materials:  crystallographic information; 3D view; checkCIF report

## Figures and Tables

**Fig. 1.**
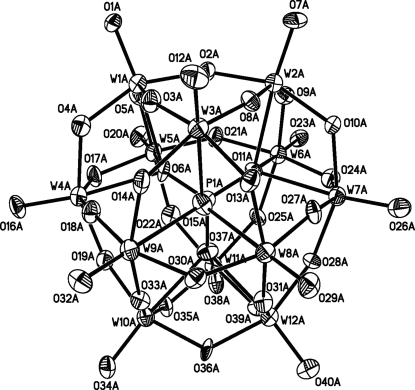
The molecular structure of one independent molecule [A] of H3[PW12O40] with 50% displacement ellipsoids. H atoms were not located.

**Fig. 2.**
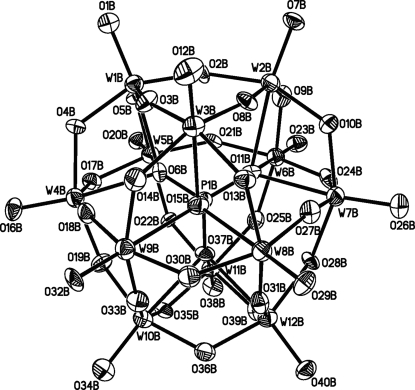
The molecular structure of the other independent molecule [B] of H3[PW12O40] with 50% displacement ellipsoids. H atoms were not located.

**Fig. 3.**
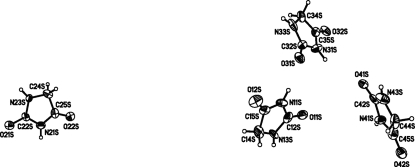
Four crystallographically independent molecules of Hydantoin. Thermal ellipsoids are drawn at the 50% probability level.

**Fig. 4.**
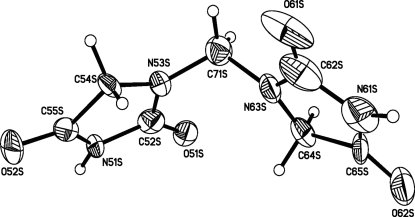
The dimerized molecule of hydantoin. Thermal ellipsoids are given at the 50% probability level.

## supplementary crystallographic information

### Comment

Hydantions, are biologically active compounds. The profound interest in different hydantoin derivatives stems from the well established medical applications of some Hydantoin derivatives as antiepileptic drugs (Micali *et al.*, 1999). Polyoxometalates (POMs) have been attracting extensive interest in solid-state materials chemistry due to their potential applications in medicine, as well as the richness of their structures (Yanagie *et al.*, 2006). Recently, an important advance in polyoxometalate chemistry is the modification of polyoxometalates with various organic ligands and transition metal complexes to obtain organic–inorganic hybrid materials (Guangzhe *et al.*, 2008). Here we report the crystal structure the title hybrid containing hydantoin.

The title compound consists of hydantoin and a dimerized species of hydantoin which crystallize with H_3_[PW_12_O_40_] and 21.5 solvent water molecules. The asymmetric unit contains two independent H_3_[PW_12_O_40_] molecules (labeled with A and B in Figs. 1 & 2). The [PW_12_O_40_]^-3 ^anion (Keggin structure) consists of a central PO_4_ tetrahedron that surrounded by twelve WO_6_ octahedrons (Keggin, 1933). In molecule A of H_3_PW_12_O_40_, all W–Ot(terminal) bonds are essentially equal, ranging from 1.697 (13)–1.724 (12) Å (mean 1.709 Å). In molecule B these values are 1.684 (11)–1.736 (13) A° mean (1.710 Å). The P–O distances in the two independendent molecules of H_3_PW_12_O_40_ are equal within experimental error ranging from 1.519 (11)-1.556 (12) A° (mean 1.538 A°) for molecule A and in the range 1.526 (12)–1.537 (12) A° (mean 1.530 A°) for molecule B. These quantities are similar to the W–Ot and P–O distances of a related Keggin structure which are 1.7041 (11) and 1.5305 (11) A° (Lebeden *et al.*, 1969). In the two independent molecules of H_3_PW_12_O_40_ the O—P—O angles are close to the ideal 109.5° based sp^3^ hybridization principles. The four independent molecules of hydantoin are shown in (Fig. 3). The pseudo-dimerized species of hydantoin is shown in (Fig. 4).

### Experimental

A solution of Hydantoin (0.06 g, 0.60 mmol) in 5 ml of 1.00 M HCl was added to a stirred solution of H_3_[PW_12_O_40_] (0.28 g, 0.10 mmol) in 10 ml H_2_O and stirred at room temperature for 2 h. After stirring, it was filtered. The filtrate was allowed to evaporate slowly at room temperature. Good-quality white prism crystals were obtained after 4 days which were filtered off and washed with water, then dried at ambient temperature.

### Refinement

Neither the hydrogen atoms of the water molecules not the hydrogen atoms in the two crystallographically independent H_3_W_12_PO_40_ molecules could be located and were not included in the refinement. These H atoms are included in the molecular formula. The hydrogen atoms in the organic molecules (four independent hydantoin molecules and the dimerized molecule) were placed geometrically [C-H = 0.99 & N-H = 0.88 Å] and refined in a riding-model approximation with the U_iso_(H) parameters equal to 1.2U_eq_(C,N). The crystals used was an inversion twin with the ratio of twin components being 0.492 (11):0.508 (11).

### Crystal data

**Table:**

2{H_3_[PW_12_O_40_]}·4C_3_H_4_N_2_O_2_·C_7_H_8_N_4_O_4_·21.5H_2_O	*Z* = 1
*M**_r_* = 6760.23	*F*(000) = 2985
Triclinic, *P*1	*D*_x_ = 4.083 Mg m^−^^3^
Hall symbol: P 1	Mo *K*α radiation, λ = 0.71073 Å
*a* = 12.7271 (6) Å	Cell parameters from 5550 reflections
*b* = 13.3286 (7) Å	θ = 2.3–27.8°
*c* = 18.9177 (9) Å	µ = 25.15 mm^−^^1^
α = 71.328 (1)°	*T* = 100 K
β = 81.316 (1)°	Prism, colourless
γ = 64.751 (1)°	0.31 × 0.17 × 0.08 mm
*V* = 2749.3 (2) Å^3^	

### Data collection

**Table:**

Bruker SMART APEXII CCD diffractometer	25728 independent reflections
Radiation source: fine-focus sealed tube	22772 reflections with *I* > 2σ(*I*)
graphite	*R*_int_ = 0.048
φ and ω scans	θ_max_ = 27.9°, θ_min_ = 1.8°
Absorption correction: integration (*SADABS*; Bruker, 2005)	*h* = −16→16
*T*_min_ = 0.031, *T*_max_ = 0.215	*k* = −17→17
51543 measured reflections	*l* = −24→24

### Refinement

**Table:**

Refinement on *F*^2^	Secondary atom site location: difference Fourier map
Least-squares matrix: full	Hydrogen site location: inferred from neighbouring sites
*R*[*F*^2^ > 2σ(*F*^2^)] = 0.043	H-atom parameters constrained
*wR*(*F*^2^) = 0.102	*w* = 1/[σ^2^(*F*_o_^2^) + (0.0474*P*)^2^] where *P* = (*F*_o_^2^ + 2*F*_c_^2^)/3
*S* = 1.00	(Δ/σ)_max_ = 0.002
25728 reflections	Δρ_max_ = 2.44 e Å^−^^3^
1577 parameters	Δρ_min_ = −1.32 e Å^−^^3^
45 restraints	Absolute structure: Flack (1983), 12649 Friedel pairs
Primary atom site location: structure-invariant direct methods	Flack parameter: 0.492 (11)

### Special details

**Table:**

Geometry. All esds (except the esd in the dihedral angle between two l.s. planes) are estimated using the full covariance matrix. The cell esds are taken into account individually in the estimation of esds in distances, angles and torsion angles; correlations between esds in cell parameters are only used when they are defined by crystal symmetry. An approximate (isotropic) treatment of cell esds is used for estimating esds involving l.s. planes.
Refinement. Refinement of *F*^2^ against ALL reflections. The weighted *R*-factor wR and goodness of fit *S* are based on *F*^2^, conventional *R*-factors *R* are based on *F*, with *F* set to zero for negative *F*^2^. The threshold expression of *F*^2^ > σ(*F*^2^) is used only for calculating *R*-factors(gt) etc. and is not relevant to the choice of reflections for refinement. *R*-factors based on *F*^2^ are statistically about twice as large as those based on *F*, and *R*- factors based on ALL data will be even larger.

### Fractional atomic coordinates and isotropic or equivalent isotropic displacement parameters (Å^2^)

**Table:**

	*x*	*y*	*z*	*U*_iso_*/*U*_eq_	Occ. (<1)
W1A	0.53978 (5)	0.04182 (6)	0.09204 (4)	0.02518 (14)	
W2A	0.49583 (6)	−0.05828 (6)	0.29651 (4)	0.02500 (13)	
W3A	0.79195 (5)	−0.15553 (6)	0.21328 (4)	0.02515 (14)	
W4A	0.66181 (5)	0.23803 (6)	0.02094 (4)	0.02508 (14)	
W5A	0.39098 (5)	0.31883 (6)	0.10253 (4)	0.02576 (14)	
W6A	0.35353 (5)	0.21981 (6)	0.30717 (4)	0.02537 (14)	
W7A	0.59046 (5)	0.03417 (6)	0.41337 (4)	0.02468 (13)	
W8A	0.88463 (5)	−0.05713 (6)	0.32745 (4)	0.02459 (13)	
W9A	0.91794 (5)	0.03733 (6)	0.13866 (4)	0.02505 (14)	
W10A	0.76520 (5)	0.33957 (6)	0.14672 (4)	0.02490 (13)	
W11A	0.49291 (5)	0.42650 (6)	0.22420 (4)	0.02525 (14)	
W12A	0.72596 (5)	0.24394 (6)	0.33558 (4)	0.02421 (13)	
P1A	0.6346 (4)	0.1357 (4)	0.2185 (3)	0.0210 (7)	
O1A	0.5043 (10)	−0.0312 (11)	0.0463 (6)	0.027 (2)	
O2A	0.4792 (9)	−0.0003 (12)	0.1913 (6)	0.028 (3)	
O3A	0.6879 (10)	−0.0700 (11)	0.1320 (7)	0.030 (3)	
O4A	0.6155 (10)	0.1218 (10)	0.0163 (7)	0.028 (2)	
O5A	0.4081 (9)	0.1879 (11)	0.0764 (6)	0.028 (2)	
O6A	0.5818 (9)	0.1684 (10)	0.1433 (5)	0.023 (2)	
O7A	0.4484 (11)	−0.1673 (11)	0.3169 (7)	0.033 (3)	
O8A	0.6589 (9)	−0.1396 (10)	0.2741 (6)	0.024 (2)	
O9A	0.3615 (9)	0.0727 (11)	0.3080 (7)	0.028 (3)	
O10A	0.5461 (11)	−0.0725 (11)	0.3930 (6)	0.028 (2)	
O11A	0.5539 (9)	0.0982 (9)	0.2811 (6)	0.022 (2)	
O12A	0.8423 (11)	−0.2951 (11)	0.2089 (8)	0.036 (3)	
O13A	0.8779 (9)	−0.1804 (10)	0.2968 (6)	0.025 (2)	
O14A	0.9038 (10)	−0.1018 (11)	0.1500 (6)	0.028 (3)	
O15A	0.7551 (9)	0.0322 (10)	0.2227 (6)	0.025 (2)	
O16A	0.7020 (10)	0.2876 (11)	−0.0693 (6)	0.031 (3)	
O17A	0.5005 (10)	0.3366 (12)	0.0200 (6)	0.030 (3)	
O18A	0.8050 (10)	0.1118 (11)	0.0641 (6)	0.028 (2)	
O19A	0.6965 (10)	0.3232 (11)	0.0701 (7)	0.027 (2)	
O20A	0.2635 (11)	0.4191 (11)	0.0612 (7)	0.033 (3)	
O21A	0.3442 (10)	0.2571 (11)	0.2004 (6)	0.029 (3)	
O22A	0.4415 (10)	0.4046 (11)	0.1427 (7)	0.030 (3)	
O23A	0.2126 (10)	0.2850 (11)	0.3334 (7)	0.027 (2)	
O24A	0.4353 (10)	0.1437 (10)	0.3981 (7)	0.028 (2)	
O25A	0.4121 (10)	0.3361 (11)	0.2861 (7)	0.028 (3)	
O26A	0.5979 (11)	−0.0109 (12)	0.5079 (7)	0.034 (3)	
O27A	0.7438 (9)	−0.0517 (10)	0.3842 (6)	0.024 (2)	
O28A	0.6340 (9)	0.1624 (9)	0.3880 (6)	0.021 (2)	
O29A	0.9876 (10)	−0.1355 (11)	0.3949 (7)	0.031 (3)	
O30A	0.9759 (9)	−0.0293 (10)	0.2398 (6)	0.025 (2)	
O31A	0.8386 (9)	0.0975 (9)	0.3287 (6)	0.024 (2)	
O32A	1.0446 (10)	0.0166 (11)	0.0883 (7)	0.030 (3)	
O33A	0.8745 (9)	0.1850 (9)	0.1510 (6)	0.023 (2)	
O34A	0.8409 (10)	0.4202 (11)	0.0987 (6)	0.030 (3)	
O35A	0.6180 (9)	0.4525 (11)	0.1615 (6)	0.027 (2)	
O36A	0.7991 (10)	0.3118 (10)	0.2481 (6)	0.026 (2)	
O37A	0.6478 (10)	0.2407 (10)	0.2261 (7)	0.028 (2)	
O38A	0.3997 (10)	0.5648 (11)	0.2237 (7)	0.030 (3)	
O39A	0.5886 (9)	0.3808 (10)	0.3087 (7)	0.027 (2)	
O40A	0.7763 (9)	0.2702 (11)	0.4045 (7)	0.030 (3)	
W1B	0.28500 (6)	0.89573 (6)	0.78057 (4)	0.02576 (14)	
W2B	0.00805 (6)	1.06170 (6)	0.67960 (4)	0.02648 (14)	
W3B	0.28826 (6)	1.00323 (6)	0.57489 (4)	0.02721 (14)	
W4B	0.45052 (6)	0.61863 (6)	0.77374 (4)	0.02643 (14)	
W5B	0.18881 (6)	0.68177 (6)	0.86603 (4)	0.02589 (14)	
W6B	−0.08422 (6)	0.84404 (6)	0.76072 (4)	0.02613 (14)	
W7B	−0.07457 (5)	0.94697 (6)	0.57102 (4)	0.02616 (14)	
W8B	0.20857 (5)	0.88111 (6)	0.47052 (4)	0.02518 (14)	
W9B	0.46105 (5)	0.72830 (6)	0.56726 (4)	0.02652 (14)	
W10B	0.36317 (6)	0.49175 (6)	0.65709 (4)	0.02731 (14)	
W11B	0.10511 (6)	0.54496 (6)	0.75489 (4)	0.02588 (14)	
W12B	0.10685 (6)	0.64792 (6)	0.56430 (4)	0.02617 (14)	
P1B	0.1922 (4)	0.7782 (5)	0.6687 (3)	0.0219 (8)	
O1B	0.3175 (10)	0.9766 (11)	0.8217 (7)	0.029 (3)	
O2B	0.1302 (8)	0.9866 (10)	0.7499 (6)	0.025 (2)	
O3B	0.3239 (10)	0.9437 (11)	0.6800 (6)	0.027 (2)	
O4B	0.4279 (10)	0.7579 (10)	0.7941 (7)	0.028 (2)	
O5B	0.2251 (9)	0.8038 (10)	0.8653 (6)	0.023 (2)	
O6B	0.2535 (9)	0.7532 (10)	0.7399 (6)	0.025 (2)	
O7B	−0.0521 (10)	1.2020 (11)	0.6817 (7)	0.031 (3)	
O8B	0.1325 (10)	1.0612 (9)	0.6062 (6)	0.024 (2)	
O9B	−0.0850 (10)	0.9948 (11)	0.7468 (7)	0.030 (3)	
O10B	−0.0791 (11)	1.0705 (11)	0.6024 (7)	0.033 (3)	
O11B	0.0666 (10)	0.8656 (10)	0.6694 (6)	0.025 (2)	
O12B	0.3202 (13)	1.1247 (13)	0.5464 (7)	0.039 (3)	
O13B	0.2408 (10)	1.0022 (10)	0.4833 (6)	0.024 (2)	
O14B	0.4367 (10)	0.8791 (11)	0.5611 (6)	0.030 (3)	
O15B	0.2586 (10)	0.8244 (11)	0.6015 (6)	0.027 (2)	
O16B	0.5857 (10)	0.5264 (11)	0.8097 (7)	0.031 (3)	
O17B	0.3547 (10)	0.5888 (10)	0.8594 (7)	0.028 (2)	
O18B	0.4851 (9)	0.6866 (11)	0.6707 (6)	0.026 (2)	
O19B	0.4135 (11)	0.5202 (11)	0.7363 (6)	0.029 (3)	
O20B	0.1599 (10)	0.6306 (11)	0.9581 (6)	0.029 (3)	
O21B	0.0380 (10)	0.7849 (10)	0.8299 (7)	0.028 (2)	
O22B	0.1703 (9)	0.5737 (10)	0.8247 (6)	0.023 (2)	
O23B	−0.1999 (10)	0.8450 (12)	0.8189 (7)	0.031 (3)	
O24B	−0.1492 (9)	0.9040 (10)	0.6667 (7)	0.027 (2)	
O25B	−0.0219 (9)	0.6901 (11)	0.7526 (7)	0.028 (2)	
O26B	−0.1882 (11)	1.0139 (12)	0.5104 (7)	0.035 (3)	
O27B	0.0542 (9)	0.9521 (11)	0.5083 (6)	0.026 (2)	
O28B	−0.0162 (9)	0.7889 (9)	0.5745 (6)	0.023 (2)	
O29B	0.1888 (10)	0.9244 (11)	0.3778 (6)	0.029 (3)	
O30B	0.3732 (10)	0.7856 (12)	0.4814 (7)	0.032 (3)	
O31B	0.1829 (9)	0.7413 (11)	0.5027 (6)	0.027 (2)	
O32B	0.6004 (9)	0.6773 (11)	0.5336 (7)	0.029 (3)	
O33B	0.4198 (10)	0.5990 (11)	0.5911 (7)	0.029 (3)	
O34B	0.4721 (11)	0.3607 (12)	0.6525 (7)	0.034 (3)	
O35B	0.2565 (10)	0.4442 (10)	0.7303 (7)	0.028 (2)	
O36B	0.2576 (11)	0.5220 (11)	0.5830 (7)	0.031 (3)	
O37B	0.1927 (10)	0.6648 (9)	0.6643 (7)	0.027 (2)	
O38B	0.0547 (11)	0.4455 (11)	0.8109 (7)	0.033 (3)	
O39B	0.0583 (9)	0.5645 (10)	0.6580 (6)	0.027 (2)	
O40B	0.0542 (12)	0.6090 (12)	0.5041 (7)	0.035 (3)	
O11S	0.5640 (12)	0.9099 (13)	0.7449 (8)	0.041 (3)	
O12S	0.8214 (12)	0.5389 (14)	0.7586 (9)	0.053 (4)	
N11S	0.6885 (14)	0.7143 (15)	0.7709 (9)	0.038 (4)	
H11A	0.6807	0.6948	0.8198	0.045*	
C12S	0.6292 (15)	0.8277 (19)	0.7240 (12)	0.039 (4)	
N13S	0.6611 (13)	0.8208 (15)	0.6528 (8)	0.034 (3)	
H13A	0.6340	0.8791	0.6126	0.041*	
C14S	0.7414 (18)	0.711 (2)	0.6518 (12)	0.048 (5)	
H14A	0.8159	0.7131	0.6288	0.057*	
H14B	0.7117	0.6777	0.6235	0.057*	
C15S	0.7572 (14)	0.6404 (18)	0.7329 (10)	0.035 (4)	
O21S	0.2573 (10)	0.0468 (12)	0.1061 (7)	0.037 (3)	
O22S	0.0405 (11)	0.3550 (12)	0.1969 (7)	0.037 (3)	
N21S	0.1446 (14)	0.2227 (15)	0.1342 (9)	0.038 (4)	
H21A	0.1477	0.2698	0.0898	0.045*	
C22S	0.1957 (12)	0.1059 (17)	0.1513 (10)	0.030 (4)	
N23S	0.1727 (13)	0.0582 (13)	0.2205 (8)	0.029 (3)	
H23A	0.1970	−0.0166	0.2426	0.035*	
C24S	0.1022 (14)	0.1475 (18)	0.2538 (10)	0.034 (4)	
H24A	0.1404	0.1417	0.2976	0.041*	
H24B	0.0256	0.1443	0.2695	0.041*	
C25S	0.0897 (13)	0.2568 (18)	0.1922 (11)	0.033 (4)	
O31S	0.6244 (12)	0.6492 (13)	0.9293 (7)	0.042 (3)	
O32S	0.2598 (12)	0.8610 (13)	1.0077 (7)	0.041 (3)	
N31S	0.4519 (13)	0.7778 (15)	0.9624 (9)	0.039 (4)	
H31A	0.4651	0.8409	0.9433	0.047*	
C32S	0.5303 (13)	0.6685 (14)	0.9602 (9)	0.026 (3)	
N33S	0.4787 (15)	0.5935 (17)	0.9991 (9)	0.043 (4)	
H33A	0.5109	0.5179	1.0073	0.052*	
C34S	0.3622 (15)	0.6594 (17)	1.0244 (11)	0.037 (4)	
H34A	0.3023	0.6496	1.0028	0.044*	
H34B	0.3553	0.6346	1.0795	0.044*	
C35S	0.3507 (15)	0.7751 (16)	0.9983 (9)	0.032 (4)	
O41S	0.7423 (11)	0.8810 (12)	0.9700 (7)	0.037 (3)	
O42S	0.9170 (14)	1.1328 (14)	0.8526 (9)	0.051 (4)	
N41S	0.8051 (14)	1.0245 (14)	0.8993 (9)	0.037 (4)	
H41A	0.7433	1.0709	0.8718	0.045*	
C42S	0.8178 (19)	0.9196 (19)	0.9534 (10)	0.040 (4)	
N43S	0.9293 (16)	0.8672 (18)	0.9850 (10)	0.050 (5)	
H43A	0.9590	0.8011	1.0199	0.061*	
C44S	0.9844 (15)	0.948 (2)	0.9466 (13)	0.044 (5)	
H44A	1.0063	0.9743	0.9832	0.053*	
H44B	1.0555	0.9092	0.9194	0.053*	
C45S	0.9028 (18)	1.044 (2)	0.8957 (14)	0.050 (6)	
O51S	0.2438 (10)	0.8084 (12)	0.2496 (7)	0.035 (3)	
O52S	0.1195 (13)	0.6632 (15)	0.1058 (8)	0.047 (4)	
N51S	0.1783 (11)	0.7587 (13)	0.1615 (8)	0.028 (3)	
H51A	0.1888	0.8090	0.1209	0.034*	
C52S	0.1986 (15)	0.7530 (16)	0.2332 (11)	0.034 (4)	
N53S	0.1671 (12)	0.6717 (14)	0.2797 (8)	0.030 (3)	
C54S	0.1341 (15)	0.6151 (17)	0.2409 (8)	0.032 (4)	
H54A	0.0541	0.6205	0.2550	0.038*	
H54B	0.1881	0.5325	0.2508	0.038*	
C55S	0.1413 (14)	0.6801 (15)	0.1614 (9)	0.029 (4)	
O61S	0.262 (2)	0.3989 (18)	0.4493 (10)	0.072 (6)	
O62S	0.5802 (15)	0.4843 (18)	0.4446 (9)	0.064 (5)	
N61S	0.4395 (18)	0.4107 (17)	0.4572 (11)	0.052 (5)	
H61A	0.4773	0.3406	0.4863	0.062*	
C62S	0.322 (3)	0.453 (3)	0.4312 (15)	0.063 (7)	
N63S	0.3002 (13)	0.5580 (14)	0.3869 (8)	0.034 (3)	
C64S	0.3925 (17)	0.5963 (16)	0.3839 (10)	0.035 (4)	
H64A	0.3664	0.6624	0.4050	0.042*	
H64B	0.4218	0.6182	0.3322	0.042*	
C65S	0.4819 (16)	0.4899 (19)	0.4316 (10)	0.038 (4)	
C71S	0.1830 (16)	0.6370 (18)	0.3608 (11)	0.039 (4)	
H71A	0.1601	0.7077	0.3764	0.047*	
H71B	0.1292	0.5999	0.3861	0.047*	
O1W	0.5503 (12)	0.9421 (12)	0.8854 (8)	0.043 (3)	
O2W	0.4125 (12)	0.8069 (12)	0.1466 (8)	0.041 (3)	
O3W	0.3800 (17)	0.3375 (14)	0.8981 (11)	0.070 (6)	
O4W	0.9874 (11)	0.5576 (11)	0.0836 (7)	0.039 (3)	
O5W	0.2477 (15)	0.0725 (19)	0.9631 (11)	0.065 (5)	
O6W	0.9302 (13)	0.4123 (13)	0.3241 (8)	0.046 (3)	
O7W	0.7473 (16)	0.6829 (15)	0.0709 (10)	0.059 (4)	
O8W	0.6186 (14)	0.7045 (18)	0.1852 (10)	0.066 (5)	
O9W	0.7353 (16)	0.3242 (14)	0.7712 (8)	0.055 (4)	
O10W	0.6992 (16)	0.5738 (18)	0.3186 (10)	0.073 (5)	
O11W	0.3862 (17)	0.1428 (16)	0.8723 (11)	0.070 (5)	
O12W	0.1626 (15)	0.3317 (18)	0.9750 (9)	0.064 (5)	
O13W	−0.0175 (17)	0.5272 (15)	0.9526 (11)	0.071 (5)	
O14W	0.6103 (16)	0.161 (2)	0.8070 (11)	0.073 (5)	
O15W	0.9674 (18)	0.531 (2)	0.3885 (9)	0.071 (5)	
O16W	0.7427 (18)	0.332 (2)	0.5480 (11)	0.096 (8)	
O17W	0.8394 (18)	0.473 (2)	0.9142 (11)	0.113 (10)	
O18W	0.992 (3)	0.238 (3)	0.4653 (15)	0.047 (8)	0.50
O19W	0.774 (2)	0.677 (2)	0.4218 (15)	0.037 (6)	0.50
O20W	0.001 (3)	0.260 (3)	0.9504 (16)	0.047 (7)	0.50
O21W	0.759 (2)	0.434 (2)	0.6399 (13)	0.033 (5)	0.50
O22W	0.082 (2)	0.366 (3)	0.503 (2)	0.053 (9)	0.50
O23W	−0.024 (3)	0.423 (3)	0.6304 (19)	0.058 (10)	0.50
O24W	0.603 (3)	0.200 (3)	0.5587 (17)	0.053 (7)	0.50
O25W	0.436 (3)	0.141 (3)	0.685 (2)	0.066 (9)	0.50
O26W	0.086 (3)	0.191 (3)	0.840 (3)	0.092 (16)	0.50

### Atomic displacement parameters (Å^2^)

**Table:**

	*U*^11^	*U*^22^	*U*^33^	*U*^12^	*U*^13^	*U*^23^
W1A	0.0258 (3)	0.0264 (3)	0.0236 (3)	−0.0100 (3)	−0.0008 (2)	−0.0081 (3)
W2A	0.0263 (3)	0.0256 (3)	0.0238 (3)	−0.0113 (3)	−0.0002 (2)	−0.0070 (3)
W3A	0.0253 (3)	0.0229 (3)	0.0265 (3)	−0.0081 (3)	−0.0014 (2)	−0.0079 (3)
W4A	0.0264 (3)	0.0255 (3)	0.0217 (3)	−0.0093 (3)	−0.0003 (2)	−0.0064 (3)
W5A	0.0249 (3)	0.0255 (3)	0.0230 (3)	−0.0061 (3)	−0.0027 (2)	−0.0065 (3)
W6A	0.0230 (3)	0.0275 (3)	0.0239 (3)	−0.0086 (3)	−0.0004 (2)	−0.0073 (3)
W7A	0.0255 (3)	0.0261 (3)	0.0220 (3)	−0.0104 (3)	−0.0014 (2)	−0.0059 (3)
W8A	0.0235 (3)	0.0239 (3)	0.0250 (3)	−0.0079 (3)	−0.0028 (2)	−0.0066 (3)
W9A	0.0231 (3)	0.0246 (3)	0.0255 (3)	−0.0081 (3)	0.0011 (2)	−0.0075 (3)
W10A	0.0246 (3)	0.0244 (3)	0.0246 (3)	−0.0092 (3)	0.0005 (2)	−0.0072 (3)
W11A	0.0245 (3)	0.0229 (3)	0.0253 (3)	−0.0061 (3)	−0.0006 (2)	−0.0077 (3)
W12A	0.0250 (3)	0.0243 (3)	0.0233 (3)	−0.0090 (3)	−0.0015 (2)	−0.0076 (3)
P1A	0.0201 (16)	0.0196 (18)	0.0218 (18)	−0.0061 (14)	−0.0013 (13)	−0.0064 (15)
O1A	0.037 (6)	0.032 (7)	0.018 (5)	−0.019 (5)	−0.007 (4)	−0.004 (5)
O2A	0.023 (5)	0.044 (7)	0.021 (5)	−0.018 (5)	−0.003 (4)	−0.008 (5)
O3A	0.029 (6)	0.031 (7)	0.026 (6)	−0.010 (5)	−0.004 (5)	−0.006 (5)
O4A	0.034 (6)	0.019 (6)	0.026 (6)	−0.006 (5)	−0.003 (5)	−0.004 (5)
O5A	0.022 (5)	0.029 (6)	0.029 (6)	−0.012 (5)	−0.003 (4)	−0.001 (5)
O6A	0.025 (5)	0.031 (6)	0.010 (5)	−0.008 (5)	−0.001 (4)	−0.007 (4)
O7A	0.039 (6)	0.024 (6)	0.044 (7)	−0.016 (5)	−0.004 (5)	−0.014 (5)
O8A	0.020 (5)	0.024 (6)	0.027 (6)	−0.009 (5)	0.005 (4)	−0.007 (5)
O9A	0.021 (5)	0.031 (6)	0.033 (6)	−0.011 (5)	−0.006 (4)	−0.006 (5)
O10A	0.038 (6)	0.036 (7)	0.018 (5)	−0.023 (5)	−0.002 (4)	−0.006 (5)
O11A	0.024 (5)	0.018 (5)	0.025 (6)	−0.011 (4)	0.001 (4)	−0.005 (4)
O12A	0.036 (7)	0.020 (6)	0.047 (8)	−0.009 (5)	−0.006 (6)	−0.003 (6)
O13A	0.019 (5)	0.030 (6)	0.023 (5)	−0.005 (5)	−0.003 (4)	−0.010 (5)
O14A	0.027 (5)	0.038 (7)	0.017 (5)	−0.012 (5)	0.003 (4)	−0.010 (5)
O15A	0.016 (5)	0.024 (6)	0.029 (6)	−0.005 (4)	0.001 (4)	−0.003 (5)
O16A	0.033 (6)	0.035 (7)	0.020 (6)	−0.017 (5)	0.000 (4)	0.004 (5)
O17A	0.025 (5)	0.041 (7)	0.023 (6)	−0.015 (5)	0.009 (4)	−0.010 (5)
O18A	0.028 (5)	0.029 (6)	0.021 (6)	−0.009 (5)	−0.005 (4)	−0.003 (5)
O19A	0.030 (5)	0.028 (6)	0.023 (6)	−0.012 (5)	0.000 (4)	−0.007 (5)
O20A	0.039 (7)	0.023 (6)	0.027 (6)	−0.004 (5)	−0.011 (5)	−0.003 (5)
O21A	0.030 (6)	0.036 (7)	0.018 (5)	−0.012 (5)	−0.004 (4)	−0.005 (5)
O22A	0.022 (5)	0.028 (6)	0.031 (6)	−0.002 (5)	−0.005 (4)	−0.007 (5)
O23A	0.026 (5)	0.028 (6)	0.031 (6)	−0.008 (5)	0.002 (5)	−0.017 (5)
O24A	0.035 (6)	0.016 (5)	0.029 (6)	−0.006 (5)	−0.003 (5)	−0.008 (5)
O25A	0.030 (6)	0.031 (7)	0.028 (6)	−0.018 (5)	0.010 (5)	−0.009 (5)
O26A	0.034 (6)	0.038 (7)	0.039 (7)	−0.018 (6)	0.002 (5)	−0.017 (6)
O27A	0.021 (5)	0.023 (6)	0.019 (5)	−0.005 (4)	0.005 (4)	−0.003 (4)
O28A	0.022 (5)	0.018 (5)	0.026 (6)	−0.011 (4)	0.001 (4)	−0.006 (4)
O29A	0.032 (6)	0.025 (6)	0.028 (6)	−0.006 (5)	−0.009 (5)	−0.001 (5)
O30A	0.016 (5)	0.028 (6)	0.028 (6)	−0.005 (4)	−0.005 (4)	−0.009 (5)
O31A	0.025 (4)	0.014 (4)	0.026 (4)	−0.002 (3)	0.001 (3)	−0.006 (3)
O32A	0.025 (6)	0.023 (6)	0.043 (7)	−0.008 (5)	0.011 (5)	−0.017 (6)
O33A	0.020 (5)	0.017 (5)	0.026 (6)	−0.005 (4)	−0.003 (4)	−0.003 (4)
O34A	0.030 (6)	0.035 (7)	0.023 (6)	−0.012 (5)	0.005 (4)	−0.010 (5)
O35A	0.023 (5)	0.033 (7)	0.019 (5)	−0.010 (5)	0.005 (4)	−0.004 (5)
O36A	0.037 (6)	0.031 (6)	0.011 (5)	−0.012 (5)	0.004 (4)	−0.012 (5)
O37A	0.032 (6)	0.017 (5)	0.036 (7)	−0.008 (5)	−0.002 (5)	−0.013 (5)
O38A	0.026 (5)	0.029 (6)	0.025 (6)	−0.005 (5)	0.009 (4)	−0.011 (5)
O39A	0.023 (5)	0.027 (6)	0.033 (6)	−0.012 (5)	−0.001 (4)	−0.010 (5)
O40A	0.020 (5)	0.032 (7)	0.038 (7)	−0.005 (5)	0.000 (4)	−0.020 (6)
W1B	0.0282 (3)	0.0258 (3)	0.0244 (3)	−0.0108 (3)	−0.0012 (2)	−0.0084 (3)
W2B	0.0285 (3)	0.0228 (3)	0.0257 (3)	−0.0072 (3)	−0.0015 (2)	−0.0078 (3)
W3B	0.0317 (3)	0.0270 (3)	0.0244 (3)	−0.0136 (3)	0.0010 (2)	−0.0075 (3)
W4B	0.0258 (3)	0.0261 (3)	0.0255 (3)	−0.0084 (3)	−0.0016 (2)	−0.0073 (3)
W5B	0.0289 (3)	0.0263 (3)	0.0221 (3)	−0.0110 (3)	−0.0002 (2)	−0.0068 (3)
W6B	0.0258 (3)	0.0277 (4)	0.0237 (3)	−0.0093 (3)	0.0018 (2)	−0.0090 (3)
W7B	0.0254 (3)	0.0272 (3)	0.0243 (3)	−0.0084 (3)	−0.0015 (2)	−0.0080 (3)
W8B	0.0268 (3)	0.0257 (3)	0.0219 (3)	−0.0098 (3)	−0.0001 (2)	−0.0066 (3)
W9B	0.0247 (3)	0.0282 (4)	0.0251 (3)	−0.0094 (3)	0.0009 (2)	−0.0079 (3)
W10B	0.0293 (3)	0.0238 (3)	0.0273 (3)	−0.0083 (3)	0.0013 (3)	−0.0095 (3)
W11B	0.0301 (3)	0.0254 (3)	0.0234 (3)	−0.0120 (3)	0.0007 (2)	−0.0078 (3)
W12B	0.0301 (3)	0.0268 (3)	0.0229 (3)	−0.0123 (3)	0.0007 (2)	−0.0083 (3)
P1B	0.0255 (17)	0.0232 (19)	0.0173 (18)	−0.0098 (15)	−0.0009 (14)	−0.0059 (15)
O1B	0.035 (6)	0.030 (7)	0.025 (6)	−0.016 (5)	0.001 (5)	−0.007 (5)
O2B	0.014 (5)	0.029 (6)	0.023 (6)	0.004 (4)	−0.001 (4)	−0.012 (5)
O3B	0.038 (6)	0.034 (7)	0.015 (5)	−0.022 (6)	−0.001 (4)	−0.004 (5)
O4B	0.029 (6)	0.022 (6)	0.034 (6)	−0.005 (5)	−0.001 (5)	−0.016 (5)
O5B	0.029 (5)	0.027 (6)	0.020 (5)	−0.014 (5)	0.003 (4)	−0.014 (5)
O6B	0.019 (5)	0.025 (6)	0.028 (6)	−0.004 (4)	−0.007 (4)	−0.007 (5)
O7B	0.034 (6)	0.023 (6)	0.029 (6)	−0.003 (5)	0.001 (5)	−0.014 (5)
O8B	0.032 (6)	0.014 (5)	0.018 (5)	−0.003 (5)	0.001 (4)	−0.002 (4)
O9B	0.030 (6)	0.034 (7)	0.026 (6)	−0.019 (5)	0.003 (5)	−0.004 (5)
O10B	0.039 (7)	0.030 (7)	0.029 (6)	−0.008 (5)	−0.011 (5)	−0.011 (5)
O11B	0.035 (6)	0.024 (6)	0.021 (5)	−0.016 (5)	0.003 (4)	−0.010 (5)
O12B	0.060 (8)	0.047 (8)	0.019 (6)	−0.032 (7)	0.000 (6)	−0.005 (6)
O13B	0.032 (5)	0.018 (5)	0.020 (5)	−0.010 (4)	0.000 (4)	−0.004 (4)
O14B	0.027 (5)	0.038 (7)	0.026 (6)	−0.013 (5)	0.003 (4)	−0.013 (5)
O15B	0.032 (6)	0.028 (6)	0.021 (6)	−0.012 (5)	0.002 (4)	−0.010 (5)
O16B	0.028 (6)	0.025 (6)	0.038 (7)	−0.013 (5)	−0.011 (5)	0.001 (5)
O17B	0.032 (6)	0.021 (6)	0.029 (6)	−0.009 (5)	−0.001 (5)	−0.008 (5)
O18B	0.019 (5)	0.030 (6)	0.023 (6)	−0.004 (5)	0.001 (4)	−0.010 (5)
O19B	0.039 (6)	0.032 (7)	0.021 (6)	−0.018 (6)	−0.003 (5)	−0.009 (5)
O20B	0.035 (6)	0.031 (7)	0.023 (6)	−0.013 (5)	−0.001 (5)	−0.010 (5)
O21B	0.028 (5)	0.016 (5)	0.036 (7)	−0.002 (5)	−0.002 (5)	−0.013 (5)
O22B	0.027 (5)	0.025 (6)	0.021 (5)	−0.009 (5)	−0.006 (4)	−0.010 (5)
O23B	0.032 (6)	0.036 (7)	0.033 (6)	−0.017 (6)	0.010 (5)	−0.018 (6)
O24B	0.022 (5)	0.017 (5)	0.036 (6)	−0.007 (4)	0.001 (4)	−0.001 (5)
O25B	0.024 (5)	0.030 (6)	0.033 (6)	−0.013 (5)	−0.001 (4)	−0.010 (5)
O26B	0.030 (6)	0.036 (7)	0.033 (7)	−0.010 (6)	−0.007 (5)	−0.005 (6)
O27B	0.027 (5)	0.034 (7)	0.013 (5)	−0.013 (5)	0.005 (4)	−0.004 (5)
O28B	0.022 (5)	0.009 (5)	0.032 (6)	−0.005 (4)	−0.001 (4)	−0.002 (4)
O29B	0.036 (6)	0.040 (7)	0.016 (5)	−0.019 (6)	−0.001 (4)	−0.006 (5)
O30B	0.030 (6)	0.038 (7)	0.024 (6)	−0.007 (5)	0.001 (5)	−0.016 (5)
O31B	0.021 (5)	0.029 (6)	0.024 (6)	−0.007 (5)	0.000 (4)	−0.005 (5)
O32B	0.013 (4)	0.035 (6)	0.031 (5)	0.001 (4)	0.007 (4)	−0.017 (5)
O33B	0.029 (6)	0.034 (7)	0.025 (6)	−0.013 (5)	0.006 (5)	−0.011 (5)
O34B	0.038 (7)	0.030 (7)	0.026 (6)	−0.010 (6)	0.001 (5)	−0.004 (5)
O35B	0.029 (6)	0.017 (6)	0.033 (6)	−0.004 (5)	−0.002 (5)	−0.010 (5)
O36B	0.036 (6)	0.025 (6)	0.028 (6)	−0.007 (5)	−0.002 (5)	−0.010 (5)
O37B	0.037 (6)	0.013 (5)	0.031 (6)	−0.008 (5)	−0.006 (5)	−0.007 (5)
O38B	0.034 (6)	0.031 (7)	0.033 (7)	−0.014 (5)	0.007 (5)	−0.009 (5)
O39B	0.022 (5)	0.027 (6)	0.024 (6)	−0.007 (5)	−0.003 (4)	−0.002 (5)
O40B	0.048 (7)	0.030 (7)	0.025 (6)	−0.012 (6)	−0.012 (5)	−0.007 (5)
O11S	0.047 (8)	0.041 (8)	0.037 (7)	−0.023 (7)	−0.002 (6)	−0.003 (6)
O12S	0.027 (7)	0.049 (10)	0.069 (10)	−0.011 (7)	−0.003 (6)	−0.008 (8)
N11S	0.040 (8)	0.037 (9)	0.024 (7)	−0.013 (7)	−0.005 (6)	0.007 (7)
C12S	0.025 (8)	0.047 (12)	0.059 (13)	−0.026 (8)	0.003 (8)	−0.021 (10)
N13S	0.039 (8)	0.050 (10)	0.015 (6)	−0.018 (8)	−0.002 (5)	−0.011 (7)
C14S	0.039 (10)	0.076 (17)	0.047 (12)	−0.031 (11)	−0.005 (9)	−0.029 (12)
C15S	0.022 (8)	0.054 (12)	0.038 (10)	−0.027 (9)	0.002 (7)	−0.010 (9)
O21S	0.027 (6)	0.040 (8)	0.038 (7)	−0.011 (6)	0.000 (5)	−0.008 (6)
O22S	0.034 (6)	0.038 (8)	0.037 (7)	−0.013 (6)	−0.007 (5)	−0.006 (6)
N21S	0.041 (8)	0.035 (9)	0.031 (8)	−0.016 (7)	0.004 (6)	−0.003 (7)
C22S	0.013 (6)	0.044 (10)	0.040 (10)	−0.015 (7)	0.012 (6)	−0.023 (8)
N23S	0.047 (8)	0.021 (7)	0.020 (7)	−0.014 (6)	−0.007 (6)	−0.003 (6)
C24S	0.024 (8)	0.056 (12)	0.026 (8)	−0.021 (8)	−0.003 (6)	−0.011 (8)
C25S	0.016 (7)	0.044 (11)	0.047 (11)	−0.013 (7)	−0.002 (7)	−0.023 (9)
O31S	0.048 (8)	0.049 (9)	0.028 (7)	−0.017 (7)	−0.010 (6)	−0.009 (6)
O32S	0.042 (7)	0.046 (8)	0.025 (6)	−0.009 (6)	−0.004 (5)	−0.010 (6)
N31S	0.039 (8)	0.044 (10)	0.034 (8)	−0.028 (8)	−0.015 (6)	0.009 (7)
C32S	0.020 (7)	0.021 (8)	0.028 (8)	−0.003 (6)	−0.011 (6)	0.002 (6)
N33S	0.043 (9)	0.051 (11)	0.032 (8)	−0.004 (8)	0.000 (7)	−0.027 (8)
C34S	0.029 (8)	0.038 (10)	0.043 (11)	−0.004 (8)	0.008 (7)	−0.027 (9)
C35S	0.038 (9)	0.027 (9)	0.027 (8)	−0.010 (7)	−0.020 (7)	0.001 (7)
O41S	0.041 (7)	0.042 (8)	0.030 (7)	−0.020 (6)	0.012 (5)	−0.015 (6)
O42S	0.063 (10)	0.048 (9)	0.048 (9)	−0.023 (8)	0.021 (7)	−0.028 (8)
N41S	0.041 (8)	0.027 (8)	0.033 (8)	−0.010 (7)	0.006 (6)	−0.002 (7)
C42S	0.055 (12)	0.048 (12)	0.020 (8)	−0.020 (10)	0.009 (8)	−0.018 (8)
N43S	0.046 (10)	0.050 (12)	0.033 (9)	−0.006 (9)	0.000 (7)	−0.002 (8)
C44S	0.023 (8)	0.051 (13)	0.067 (14)	−0.007 (8)	0.007 (8)	−0.044 (12)
C45S	0.037 (10)	0.062 (15)	0.065 (15)	−0.023 (11)	0.023 (10)	−0.042 (13)
O51S	0.030 (6)	0.032 (7)	0.042 (7)	−0.005 (5)	−0.002 (5)	−0.020 (6)
O52S	0.057 (9)	0.068 (11)	0.042 (8)	−0.037 (8)	−0.001 (6)	−0.033 (8)
N51S	0.027 (6)	0.040 (8)	0.022 (7)	−0.012 (6)	−0.002 (5)	−0.016 (6)
C52S	0.030 (8)	0.029 (9)	0.042 (10)	−0.006 (7)	0.003 (7)	−0.019 (8)
N53S	0.032 (7)	0.038 (8)	0.025 (7)	−0.015 (6)	−0.001 (5)	−0.013 (6)
C54S	0.038 (9)	0.045 (11)	0.014 (7)	−0.023 (8)	0.005 (6)	−0.007 (7)
C55S	0.024 (7)	0.025 (8)	0.022 (8)	0.000 (6)	−0.003 (6)	0.002 (7)
O61S	0.121 (17)	0.074 (13)	0.048 (10)	−0.066 (13)	0.042 (10)	−0.035 (10)
O62S	0.061 (10)	0.077 (13)	0.036 (9)	−0.003 (9)	−0.017 (7)	−0.020 (9)
N61S	0.067 (12)	0.041 (11)	0.044 (10)	−0.011 (10)	0.007 (9)	−0.027 (9)
C62S	0.087 (19)	0.058 (17)	0.046 (14)	−0.030 (15)	0.019 (13)	−0.027 (13)
N63S	0.040 (8)	0.041 (9)	0.020 (7)	−0.012 (7)	−0.005 (6)	−0.010 (7)
C64S	0.051 (10)	0.027 (9)	0.020 (8)	−0.013 (8)	−0.001 (7)	−0.003 (7)
C65S	0.034 (7)	0.048 (9)	0.023 (7)	−0.003 (6)	−0.015 (6)	−0.010 (6)
C71S	0.038 (10)	0.037 (11)	0.036 (10)	−0.010 (9)	−0.004 (8)	−0.008 (8)
O1W	0.037 (7)	0.036 (8)	0.054 (9)	−0.011 (6)	0.009 (6)	−0.020 (7)
O2W	0.045 (7)	0.035 (7)	0.043 (8)	−0.015 (6)	−0.008 (6)	−0.009 (6)
O3W	0.096 (14)	0.035 (9)	0.085 (13)	−0.025 (9)	−0.053 (11)	−0.007 (9)
O4W	0.044 (7)	0.027 (7)	0.044 (8)	−0.009 (6)	0.014 (6)	−0.020 (6)
O5W	0.052 (9)	0.088 (14)	0.073 (12)	−0.028 (10)	0.006 (8)	−0.048 (11)
O6W	0.047 (8)	0.038 (8)	0.045 (8)	−0.012 (7)	−0.003 (6)	−0.006 (7)
O7W	0.067 (10)	0.047 (10)	0.059 (10)	−0.027 (9)	−0.017 (8)	0.002 (8)
O8W	0.047 (9)	0.076 (13)	0.072 (12)	−0.018 (9)	−0.018 (8)	−0.019 (10)
O9W	0.086 (12)	0.041 (9)	0.039 (8)	−0.029 (9)	0.006 (8)	−0.013 (7)
O10W	0.064 (11)	0.080 (14)	0.061 (11)	−0.036 (11)	−0.002 (9)	0.008 (10)
O11W	0.074 (12)	0.045 (10)	0.075 (12)	−0.011 (9)	0.008 (9)	−0.017 (9)
O12W	0.059 (10)	0.080 (13)	0.043 (9)	−0.023 (10)	−0.022 (8)	−0.003 (9)
O13W	0.085 (13)	0.041 (10)	0.078 (13)	−0.023 (10)	0.004 (10)	−0.012 (9)
O14W	0.055 (10)	0.093 (16)	0.065 (12)	−0.034 (11)	0.003 (8)	−0.011 (11)
O15W	0.090 (13)	0.103 (16)	0.046 (10)	−0.067 (13)	0.000 (9)	−0.017 (10)
O16W	0.077 (13)	0.12 (2)	0.059 (12)	0.013 (13)	−0.039 (10)	−0.035 (13)
O17W	0.068 (12)	0.107 (19)	0.056 (12)	0.008 (12)	0.003 (9)	0.051 (12)
O18W	0.062 (18)	0.043 (17)	0.041 (15)	−0.038 (15)	−0.041 (14)	0.021 (13)
O19W	0.034 (7)	0.035 (7)	0.034 (7)	−0.010 (5)	0.008 (5)	−0.006 (5)
O20W	0.049 (16)	0.054 (19)	0.044 (16)	−0.032 (15)	−0.001 (12)	−0.005 (14)
O21W	0.040 (7)	0.030 (7)	0.028 (7)	−0.010 (5)	−0.003 (4)	−0.010 (5)
O22W	0.022 (12)	0.060 (19)	0.10 (3)	−0.024 (13)	0.032 (14)	−0.056 (19)
O23W	0.07 (2)	0.08 (2)	0.07 (2)	−0.07 (2)	0.056 (18)	−0.07 (2)
O24W	0.059 (9)	0.050 (9)	0.047 (8)	−0.013 (5)	0.000 (5)	−0.022 (5)
O25W	0.070 (10)	0.063 (10)	0.062 (10)	−0.026 (6)	0.001 (5)	−0.018 (5)
O26W	0.030 (16)	0.05 (2)	0.18 (5)	0.012 (15)	−0.01 (2)	−0.04 (3)

### Geometric parameters (Å, °)

**Table:**

W1A—O1A	1.712 (11)	W6B—O9B	1.937 (13)
W1A—O4A	1.897 (12)	W6B—O11B	2.430 (11)
W1A—O3A	1.913 (12)	W7B—O26B	1.716 (12)
W1A—O5A	1.919 (12)	W7B—O27B	1.886 (10)
W1A—O2A	1.921 (11)	W7B—O28B	1.894 (10)
W1A—O6A	2.460 (11)	W7B—O10B	1.898 (12)
W2A—O7A	1.716 (11)	W7B—O24B	1.945 (11)
W2A—O9A	1.898 (12)	W7B—O11B	2.434 (11)
W2A—O2A	1.900 (11)	W8B—O29B	1.684 (11)
W2A—O8A	1.941 (11)	W8B—O13B	1.918 (11)
W2A—O10A	1.951 (11)	W8B—O31B	1.919 (12)
W2A—O11A	2.417 (10)	W8B—O27B	1.920 (11)
W3A—O12A	1.716 (13)	W8B—O30B	1.934 (12)
W3A—O8A	1.870 (10)	W8B—O15B	2.446 (11)
W3A—O3A	1.903 (11)	W9B—O32B	1.709 (10)
W3A—O13A	1.919 (10)	W9B—O14B	1.865 (13)
W3A—O14A	1.943 (11)	W9B—O30B	1.870 (12)
W3A—O15A	2.403 (12)	W9B—O18B	1.890 (11)
W4A—O16A	1.708 (11)	W9B—O33B	1.911 (12)
W4A—O19A	1.891 (12)	W9B—O15B	2.432 (12)
W4A—O17A	1.907 (12)	W10B—O34B	1.725 (13)
W4A—O4A	1.908 (12)	W10B—O33B	1.893 (12)
W4A—O18A	1.942 (11)	W10B—O19B	1.914 (11)
W4A—O6A	2.445 (10)	W10B—O36B	1.917 (12)
W5A—O20A	1.701 (12)	W10B—O35B	1.941 (12)
W5A—O5A	1.880 (13)	W10B—O37B	2.426 (12)
W5A—O21A	1.889 (11)	W11B—O38B	1.706 (12)
W5A—O22A	1.905 (12)	W11B—O22B	1.872 (10)
W5A—O17A	1.955 (11)	W11B—O35B	1.912 (11)
W5A—O6A	2.436 (11)	W11B—O25B	1.912 (12)
W6A—O23A	1.702 (11)	W11B—O39B	1.917 (11)
W6A—O24A	1.895 (12)	W11B—O37B	2.433 (12)
W6A—O25A	1.902 (11)	W12B—O40B	1.709 (12)
W6A—O9A	1.915 (13)	W12B—O31B	1.898 (11)
W6A—O21A	1.929 (11)	W12B—O28B	1.911 (11)
W6A—O11A	2.445 (11)	W12B—O36B	1.924 (12)
W7A—O26A	1.697 (13)	W12B—O39B	1.938 (11)
W7A—O24A	1.886 (12)	W12B—O37B	2.435 (11)
W7A—O10A	1.894 (12)	P1B—O15B	1.525 (12)
W7A—O27A	1.897 (11)	P1B—O11B	1.526 (13)
W7A—O28A	1.913 (10)	P1B—O6B	1.531 (11)
W7A—O11A	2.418 (11)	P1B—O37B	1.537 (12)
W8A—O29A	1.709 (11)	O11S—C12S	1.20 (3)
W8A—O31A	1.895 (11)	O12S—C15S	1.22 (2)
W8A—O30A	1.905 (11)	N11S—C15S	1.33 (3)
W8A—O27A	1.930 (10)	N11S—C12S	1.42 (3)
W8A—O13A	1.946 (12)	N11S—H11A	0.8800
W8A—O15A	2.421 (11)	C12S—N13S	1.37 (3)
W9A—O32A	1.705 (11)	N13S—C14S	1.39 (3)
W9A—O14A	1.881 (13)	N13S—H13A	0.8800
W9A—O18A	1.881 (11)	C14S—C15S	1.51 (3)
W9A—O33A	1.887 (11)	C14S—H14A	0.9900
W9A—O30A	1.942 (11)	C14S—H14B	0.9900
W9A—O15A	2.426 (11)	O21S—C22S	1.29 (2)
W10A—O34A	1.704 (12)	O22S—C25S	1.21 (2)
W10A—O35A	1.887 (11)	N21S—C25S	1.31 (2)
W10A—O36A	1.913 (10)	N21S—C22S	1.35 (2)
W10A—O19A	1.914 (12)	N21S—H21A	0.8800
W10A—O33A	1.916 (11)	C22S—N23S	1.31 (2)
W10A—O37A	2.442 (12)	N23S—C24S	1.42 (2)
W11A—O38A	1.713 (12)	N23S—H23A	0.8800
W11A—O22A	1.909 (12)	C24S—C25S	1.51 (3)
W11A—O25A	1.916 (11)	C24S—H24A	0.9900
W11A—O35A	1.923 (10)	C24S—H24B	0.9900
W11A—O39A	1.933 (11)	O31S—C32S	1.21 (2)
W11A—O37A	2.412 (12)	O32S—C35S	1.27 (2)
W12A—O40A	1.724 (12)	N31S—C35S	1.37 (2)
W12A—O28A	1.892 (10)	N31S—C32S	1.38 (2)
W12A—O39A	1.896 (12)	N31S—H31A	0.8800
W12A—O31A	1.897 (11)	C32S—N33S	1.38 (3)
W12A—O36A	1.928 (11)	N33S—C34S	1.46 (2)
W12A—O37A	2.449 (12)	N33S—H33A	0.8800
P1A—O6A	1.519 (11)	C34S—C35S	1.41 (3)
P1A—O37A	1.529 (12)	C34S—H34A	0.9900
P1A—O11A	1.549 (11)	C34S—H34B	0.9900
P1A—O15A	1.556 (12)	O41S—C42S	1.23 (2)
W1B—O1B	1.718 (12)	O42S—C45S	1.28 (3)
W1B—O3B	1.865 (11)	N41S—C45S	1.36 (3)
W1B—O2B	1.882 (10)	N41S—C42S	1.40 (3)
W1B—O4B	1.933 (12)	N41S—H41A	0.8800
W1B—O5B	1.954 (11)	C42S—N43S	1.42 (3)
W1B—O6B	2.458 (12)	N43S—C44S	1.47 (3)
W2B—O7B	1.703 (12)	N43S—H43A	0.8800
W2B—O9B	1.880 (11)	C44S—C45S	1.42 (3)
W2B—O2B	1.907 (10)	C44S—H44A	0.9900
W2B—O10B	1.909 (11)	C44S—H44B	0.9900
W2B—O8B	1.942 (11)	O51S—C52S	1.24 (2)
W2B—O11B	2.458 (11)	O52S—C55S	1.24 (2)
W3B—O12B	1.736 (13)	N51S—C55S	1.32 (2)
W3B—O8B	1.873 (11)	N51S—C52S	1.39 (2)
W3B—O13B	1.925 (11)	N51S—H51A	0.8800
W3B—O3B	1.936 (10)	C52S—N53S	1.33 (2)
W3B—O14B	1.958 (12)	N53S—C54S	1.42 (2)
W3B—O15B	2.454 (12)	N53S—C71S	1.47 (2)
W4B—O16B	1.717 (11)	C54S—C55S	1.49 (2)
W4B—O4B	1.909 (11)	C54S—H54A	0.9900
W4B—O19B	1.914 (12)	C54S—H54B	0.9900
W4B—O17B	1.917 (12)	O61S—C62S	1.21 (3)
W4B—O18B	1.940 (11)	O62S—C65S	1.28 (3)
W4B—O6B	2.414 (10)	N61S—C65S	1.31 (3)
W5B—O20B	1.701 (12)	N61S—C62S	1.46 (4)
W5B—O5B	1.867 (11)	N61S—H61A	0.8800
W5B—O21B	1.894 (11)	C62S—N63S	1.31 (3)
W5B—O17B	1.948 (12)	N63S—C64S	1.45 (2)
W5B—O22B	1.951 (11)	N63S—C71S	1.46 (2)
W5B—O6B	2.429 (12)	C64S—C65S	1.49 (3)
W6B—O23B	1.698 (11)	C64S—H64A	0.9900
W6B—O24B	1.859 (12)	C64S—H64B	0.9900
W6B—O25B	1.908 (12)	C71S—H71A	0.9900
W6B—O21B	1.913 (12)	C71S—H71B	0.9900
			
O1A—W1A—O4A	102.7 (5)	O19B—W4B—O6B	83.8 (5)
O1A—W1A—O3A	101.8 (6)	O17B—W4B—O6B	73.5 (4)
O4A—W1A—O3A	89.5 (5)	O18B—W4B—O6B	83.5 (4)
O1A—W1A—O5A	103.5 (5)	O20B—W5B—O5B	102.0 (5)
O4A—W1A—O5A	86.1 (5)	O20B—W5B—O21B	101.3 (6)
O3A—W1A—O5A	154.6 (5)	O5B—W5B—O21B	91.8 (5)
O1A—W1A—O2A	103.4 (5)	O20B—W5B—O17B	101.7 (5)
O4A—W1A—O2A	154.0 (5)	O5B—W5B—O17B	88.3 (5)
O3A—W1A—O2A	86.0 (5)	O21B—W5B—O17B	156.5 (5)
O5A—W1A—O2A	87.1 (5)	O20B—W5B—O22B	101.3 (5)
O1A—W1A—O6A	172.5 (5)	O5B—W5B—O22B	156.6 (5)
O4A—W1A—O6A	72.0 (4)	O21B—W5B—O22B	85.8 (5)
O3A—W1A—O6A	83.6 (5)	O17B—W5B—O22B	84.9 (5)
O5A—W1A—O6A	71.3 (4)	O20B—W5B—O6B	172.5 (5)
O2A—W1A—O6A	82.0 (4)	O5B—W5B—O6B	73.3 (4)
O7A—W2A—O9A	104.5 (5)	O21B—W5B—O6B	84.9 (4)
O7A—W2A—O2A	101.4 (6)	O17B—W5B—O6B	72.6 (4)
O9A—W2A—O2A	89.9 (5)	O22B—W5B—O6B	83.3 (4)
O7A—W2A—O8A	100.9 (5)	O23B—W6B—O24B	102.9 (6)
O9A—W2A—O8A	154.7 (5)	O23B—W6B—O25B	101.2 (6)
O2A—W2A—O8A	84.9 (5)	O24B—W6B—O25B	91.5 (5)
O7A—W2A—O10A	103.1 (6)	O23B—W6B—O21B	101.7 (6)
O9A—W2A—O10A	87.8 (5)	O24B—W6B—O21B	155.3 (5)
O2A—W2A—O10A	155.3 (5)	O25B—W6B—O21B	84.9 (5)
O8A—W2A—O10A	86.7 (5)	O23B—W6B—O9B	103.1 (6)
O7A—W2A—O11A	173.6 (5)	O24B—W6B—O9B	86.9 (5)
O9A—W2A—O11A	71.9 (4)	O25B—W6B—O9B	155.4 (5)
O2A—W2A—O11A	84.1 (5)	O21B—W6B—O9B	86.4 (5)
O8A—W2A—O11A	82.9 (4)	O23B—W6B—O11B	173.2 (5)
O10A—W2A—O11A	71.8 (4)	O24B—W6B—O11B	72.4 (4)
O12A—W3A—O8A	103.2 (6)	O25B—W6B—O11B	84.0 (4)
O12A—W3A—O3A	102.8 (6)	O21B—W6B—O11B	82.9 (5)
O8A—W3A—O3A	86.0 (5)	O9B—W6B—O11B	72.1 (4)
O12A—W3A—O13A	99.9 (6)	O26B—W7B—O27B	103.5 (5)
O8A—W3A—O13A	89.9 (5)	O26B—W7B—O28B	104.2 (6)
O3A—W3A—O13A	157.2 (5)	O27B—W7B—O28B	86.3 (5)
O12A—W3A—O14A	102.0 (6)	O26B—W7B—O10B	102.8 (6)
O8A—W3A—O14A	154.8 (5)	O27B—W7B—O10B	89.9 (5)
O3A—W3A—O14A	87.2 (5)	O28B—W7B—O10B	152.9 (5)
O13A—W3A—O14A	87.1 (5)	O26B—W7B—O24B	102.5 (5)
O12A—W3A—O15A	170.3 (5)	O27B—W7B—O24B	153.9 (5)
O8A—W3A—O15A	83.7 (4)	O28B—W7B—O24B	86.1 (5)
O3A—W3A—O15A	84.1 (5)	O10B—W7B—O24B	85.7 (5)
O13A—W3A—O15A	73.1 (4)	O26B—W7B—O11B	171.8 (5)
O14A—W3A—O15A	71.4 (5)	O27B—W7B—O11B	83.1 (4)
O16A—W4A—O19A	102.6 (6)	O28B—W7B—O11B	80.9 (4)
O16A—W4A—O17A	101.3 (5)	O10B—W7B—O11B	72.0 (4)
O19A—W4A—O17A	91.3 (5)	O24B—W7B—O11B	71.0 (4)
O16A—W4A—O4A	102.5 (6)	O29B—W8B—O13B	101.6 (5)
O19A—W4A—O4A	154.7 (5)	O29B—W8B—O31B	102.9 (5)
O17A—W4A—O4A	86.8 (5)	O13B—W8B—O31B	155.5 (5)
O16A—W4A—O18A	102.9 (5)	O29B—W8B—O27B	102.6 (5)
O19A—W4A—O18A	84.9 (5)	O13B—W8B—O27B	88.1 (5)
O17A—W4A—O18A	155.7 (5)	O31B—W8B—O27B	85.5 (5)
O4A—W4A—O18A	86.5 (5)	O29B—W8B—O30B	103.9 (6)
O16A—W4A—O6A	172.4 (5)	O13B—W8B—O30B	87.3 (5)
O19A—W4A—O6A	83.1 (4)	O31B—W8B—O30B	87.9 (5)
O17A—W4A—O6A	73.3 (4)	O27B—W8B—O30B	153.5 (5)
O4A—W4A—O6A	72.2 (4)	O29B—W8B—O15B	172.8 (5)
O18A—W4A—O6A	82.4 (4)	O13B—W8B—O15B	73.3 (4)
O20A—W5A—O5A	101.7 (5)	O31B—W8B—O15B	82.4 (4)
O20A—W5A—O21A	103.2 (6)	O27B—W8B—O15B	82.6 (4)
O5A—W5A—O21A	90.7 (5)	O30B—W8B—O15B	71.1 (4)
O20A—W5A—O22A	103.5 (5)	O32B—W9B—O14B	101.2 (6)
O5A—W5A—O22A	154.5 (5)	O32B—W9B—O30B	102.6 (5)
O21A—W5A—O22A	87.5 (5)	O14B—W9B—O30B	89.5 (5)
O20A—W5A—O17A	100.5 (6)	O32B—W9B—O18B	101.7 (5)
O5A—W5A—O17A	85.0 (5)	O14B—W9B—O18B	88.7 (5)
O21A—W5A—O17A	156.3 (5)	O30B—W9B—O18B	155.6 (5)
O22A—W5A—O17A	86.6 (5)	O32B—W9B—O33B	103.7 (6)
O20A—W5A—O6A	171.2 (5)	O14B—W9B—O33B	155.1 (5)
O5A—W5A—O6A	72.4 (4)	O30B—W9B—O33B	86.0 (5)
O21A—W5A—O6A	83.7 (4)	O18B—W9B—O33B	85.5 (5)
O22A—W5A—O6A	82.1 (4)	O32B—W9B—O15B	172.3 (5)
O17A—W5A—O6A	72.8 (4)	O14B—W9B—O15B	73.3 (4)
O23A—W6A—O24A	104.4 (5)	O30B—W9B—O15B	72.4 (4)
O23A—W6A—O25A	104.3 (5)	O18B—W9B—O15B	83.7 (4)
O24A—W6A—O25A	90.0 (5)	O33B—W9B—O15B	82.0 (5)
O23A—W6A—O9A	101.4 (5)	O34B—W10B—O33B	102.2 (6)
O24A—W6A—O9A	87.6 (5)	O34B—W10B—O19B	102.3 (6)
O25A—W6A—O9A	154.0 (5)	O33B—W10B—O19B	86.6 (5)
O23A—W6A—O21A	102.8 (5)	O34B—W10B—O36B	102.4 (6)
O24A—W6A—O21A	152.7 (5)	O33B—W10B—O36B	90.6 (5)
O25A—W6A—O21A	85.6 (5)	O19B—W10B—O36B	155.2 (5)
O9A—W6A—O21A	84.7 (5)	O34B—W10B—O35B	102.2 (5)
O23A—W6A—O11A	171.1 (5)	O33B—W10B—O35B	155.4 (5)
O24A—W6A—O11A	71.4 (4)	O19B—W10B—O35B	85.9 (5)
O25A—W6A—O11A	83.7 (4)	O36B—W10B—O35B	86.5 (5)
O9A—W6A—O11A	71.0 (4)	O34B—W10B—O37B	172.6 (5)
O21A—W6A—O11A	81.4 (4)	O33B—W10B—O37B	83.6 (5)
O26A—W7A—O24A	101.9 (6)	O19B—W10B—O37B	82.4 (5)
O26A—W7A—O10A	102.6 (5)	O36B—W10B—O37B	72.8 (5)
O24A—W7A—O10A	88.6 (5)	O35B—W10B—O37B	72.2 (4)
O26A—W7A—O27A	102.4 (5)	O38B—W11B—O22B	101.6 (5)
O24A—W7A—O27A	155.6 (5)	O38B—W11B—O35B	100.1 (6)
O10A—W7A—O27A	87.5 (5)	O22B—W11B—O35B	89.8 (5)
O26A—W7A—O28A	102.3 (5)	O38B—W11B—O25B	103.6 (5)
O24A—W7A—O28A	87.1 (5)	O22B—W11B—O25B	86.0 (5)
O10A—W7A—O28A	155.1 (5)	O35B—W11B—O25B	156.2 (5)
O27A—W7A—O28A	86.4 (5)	O38B—W11B—O39B	102.2 (6)
O26A—W7A—O11A	172.2 (5)	O22B—W11B—O39B	156.2 (5)
O24A—W7A—O11A	72.1 (4)	O35B—W11B—O39B	87.4 (5)
O10A—W7A—O11A	72.7 (4)	O25B—W11B—O39B	87.1 (5)
O27A—W7A—O11A	83.7 (4)	O38B—W11B—O37B	171.0 (5)
O28A—W7A—O11A	82.7 (4)	O22B—W11B—O37B	83.8 (4)
O29A—W8A—O31A	103.4 (5)	O35B—W11B—O37B	72.5 (5)
O29A—W8A—O30A	102.3 (5)	O25B—W11B—O37B	83.8 (4)
O31A—W8A—O30A	89.5 (5)	O39B—W11B—O37B	72.8 (4)
O29A—W8A—O27A	101.9 (5)	O40B—W12B—O31B	105.1 (6)
O31A—W8A—O27A	86.3 (5)	O40B—W12B—O28B	104.2 (6)
O30A—W8A—O27A	155.8 (4)	O31B—W12B—O28B	86.5 (5)
O29A—W8A—O13A	100.8 (5)	O40B—W12B—O36B	100.1 (6)
O31A—W8A—O13A	155.7 (5)	O31B—W12B—O36B	87.5 (5)
O30A—W8A—O13A	87.7 (5)	O28B—W12B—O36B	155.7 (5)
O27A—W8A—O13A	86.5 (5)	O40B—W12B—O39B	99.3 (6)
O29A—W8A—O15A	171.6 (5)	O31B—W12B—O39B	155.5 (5)
O31A—W8A—O15A	83.8 (4)	O28B—W12B—O39B	89.6 (5)
O30A—W8A—O15A	73.1 (4)	O36B—W12B—O39B	86.3 (5)
O27A—W8A—O15A	82.8 (4)	O40B—W12B—O37B	169.0 (5)
O13A—W8A—O15A	72.3 (4)	O31B—W12B—O37B	83.0 (4)
O32A—W9A—O14A	101.0 (5)	O28B—W12B—O37B	83.4 (4)
O32A—W9A—O18A	102.8 (6)	O36B—W12B—O37B	72.5 (5)
O14A—W9A—O18A	88.8 (5)	O39B—W12B—O37B	72.5 (4)
O32A—W9A—O33A	103.0 (5)	O15B—P1B—O11B	110.4 (7)
O14A—W9A—O33A	156.0 (5)	O15B—P1B—O6B	108.7 (7)
O18A—W9A—O33A	86.3 (5)	O11B—P1B—O6B	110.7 (7)
O32A—W9A—O30A	100.9 (5)	O15B—P1B—O37B	109.4 (7)
O14A—W9A—O30A	87.1 (5)	O11B—P1B—O37B	108.8 (7)
O18A—W9A—O30A	156.3 (5)	O6B—P1B—O37B	108.8 (7)
O33A—W9A—O30A	88.1 (5)	W1B—O2B—W2B	154.2 (6)
O32A—W9A—O15A	170.1 (5)	W1B—O3B—W3B	152.4 (6)
O14A—W9A—O15A	71.8 (4)	W4B—O4B—W1B	125.6 (6)
O18A—W9A—O15A	84.1 (4)	W5B—O5B—W1B	126.8 (6)
O33A—W9A—O15A	84.3 (4)	P1B—O6B—W4B	126.7 (7)
O30A—W9A—O15A	72.4 (4)	P1B—O6B—W5B	125.1 (6)
O34A—W10A—O35A	103.1 (5)	W4B—O6B—W5B	89.6 (4)
O34A—W10A—O36A	102.3 (5)	P1B—O6B—W1B	125.8 (7)
O35A—W10A—O36A	88.3 (5)	W4B—O6B—W1B	89.1 (3)
O34A—W10A—O19A	103.7 (5)	W5B—O6B—W1B	88.7 (4)
O35A—W10A—O19A	87.2 (5)	W3B—O8B—W2B	152.5 (6)
O36A—W10A—O19A	153.9 (5)	W2B—O9B—W6B	126.9 (7)
O34A—W10A—O33A	102.5 (5)	W7B—O10B—W2B	127.9 (7)
O35A—W10A—O33A	154.2 (5)	P1B—O11B—W6B	126.8 (6)
O36A—W10A—O33A	89.2 (5)	P1B—O11B—W7B	127.0 (6)
O19A—W10A—O33A	83.9 (5)	W6B—O11B—W7B	89.1 (4)
O34A—W10A—O37A	172.5 (5)	P1B—O11B—W2B	124.5 (6)
O35A—W10A—O37A	71.7 (4)	W6B—O11B—W2B	88.6 (4)
O36A—W10A—O37A	72.6 (4)	W7B—O11B—W2B	88.7 (4)
O19A—W10A—O37A	81.6 (4)	W8B—O13B—W3B	125.3 (6)
O33A—W10A—O37A	83.1 (4)	W9B—O14B—W3B	126.5 (6)
O38A—W11A—O22A	103.2 (5)	P1B—O15B—W9B	127.2 (7)
O38A—W11A—O25A	102.7 (5)	P1B—O15B—W8B	126.0 (6)
O22A—W11A—O25A	85.4 (5)	W9B—O15B—W8B	88.7 (4)
O38A—W11A—O35A	101.5 (5)	P1B—O15B—W3B	125.6 (7)
O22A—W11A—O35A	88.8 (5)	W9B—O15B—W3B	88.7 (4)
O25A—W11A—O35A	155.8 (5)	W8B—O15B—W3B	88.3 (4)
O38A—W11A—O39A	101.5 (5)	W4B—O17B—W5B	124.0 (6)
O22A—W11A—O39A	155.3 (5)	W9B—O18B—W4B	151.3 (6)
O25A—W11A—O39A	88.2 (5)	W10B—O19B—W4B	150.4 (7)
O35A—W11A—O39A	87.3 (5)	W5B—O21B—W6B	152.0 (7)
O38A—W11A—O37A	171.1 (5)	W11B—O22B—W5B	150.5 (6)
O22A—W11A—O37A	82.8 (5)	W6B—O24B—W7B	127.4 (6)
O25A—W11A—O37A	84.1 (5)	W6B—O25B—W11B	151.6 (6)
O35A—W11A—O37A	71.8 (5)	W7B—O27B—W8B	152.6 (7)
O39A—W11A—O37A	72.8 (5)	W7B—O28B—W12B	151.9 (6)
O40A—W12A—O28A	103.9 (5)	W9B—O30B—W8B	127.4 (6)
O40A—W12A—O39A	100.6 (5)	W12B—O31B—W8B	151.6 (7)
O28A—W12A—O39A	89.3 (5)	W10B—O33B—W9B	152.6 (7)
O40A—W12A—O31A	104.4 (5)	W11B—O35B—W10B	125.5 (6)
O28A—W12A—O31A	85.4 (5)	W10B—O36B—W12B	125.4 (7)
O39A—W12A—O31A	155.1 (5)	P1B—O37B—W10B	126.2 (7)
O40A—W12A—O36A	101.2 (5)	P1B—O37B—W11B	126.2 (7)
O28A—W12A—O36A	154.8 (5)	W10B—O37B—W11B	89.6 (4)
O39A—W12A—O36A	87.4 (5)	P1B—O37B—W12B	124.6 (6)
O31A—W12A—O36A	87.2 (5)	W10B—O37B—W12B	89.2 (4)
O40A—W12A—O37A	170.4 (5)	W11B—O37B—W12B	89.4 (4)
O28A—W12A—O37A	83.0 (4)	W11B—O39B—W12B	125.3 (6)
O39A—W12A—O37A	72.5 (4)	C15S—N11S—C12S	112.8 (16)
O31A—W12A—O37A	82.6 (4)	C15S—N11S—H11A	123.6
O36A—W12A—O37A	72.2 (4)	C12S—N11S—H11A	123.6
O6A—P1A—O37A	108.7 (7)	O11S—C12S—N13S	129 (2)
O6A—P1A—O11A	109.0 (6)	O11S—C12S—N11S	125 (2)
O37A—P1A—O11A	110.7 (7)	N13S—C12S—N11S	105.4 (18)
O6A—P1A—O15A	108.8 (7)	C12S—N13S—C14S	111.8 (17)
O37A—P1A—O15A	110.7 (7)	C12S—N13S—H13A	124.1
O11A—P1A—O15A	109.1 (6)	C14S—N13S—H13A	124.1
W2A—O2A—W1A	151.9 (6)	N13S—C14S—C15S	104.9 (17)
W3A—O3A—W1A	150.8 (7)	N13S—C14S—H14A	110.8
W1A—O4A—W4A	127.5 (7)	C15S—C14S—H14A	110.8
W5A—O5A—W1A	127.9 (6)	N13S—C14S—H14B	110.8
P1A—O6A—W5A	125.7 (6)	C15S—C14S—H14B	110.8
P1A—O6A—W4A	126.5 (6)	H14A—C14S—H14B	108.8
W5A—O6A—W4A	89.0 (3)	O12S—C15S—N11S	127.1 (19)
P1A—O6A—W1A	126.7 (7)	O12S—C15S—C14S	128 (2)
W5A—O6A—W1A	88.4 (3)	N11S—C15S—C14S	105.0 (18)
W4A—O6A—W1A	88.2 (3)	C25S—N21S—C22S	111.3 (16)
W3A—O8A—W2A	152.5 (7)	C25S—N21S—H21A	124.3
W2A—O9A—W6A	127.6 (6)	C22S—N21S—H21A	124.3
W7A—O10A—W2A	125.4 (6)	O21S—C22S—N23S	123.2 (18)
P1A—O11A—W2A	126.1 (6)	O21S—C22S—N21S	125.8 (17)
P1A—O11A—W7A	125.1 (6)	N23S—C22S—N21S	111.1 (15)
W2A—O11A—W7A	89.9 (4)	C22S—N23S—C24S	108.6 (15)
P1A—O11A—W6A	126.1 (6)	C22S—N23S—H23A	125.7
W2A—O11A—W6A	89.4 (3)	C24S—N23S—H23A	125.7
W7A—O11A—W6A	88.6 (4)	N23S—C24S—C25S	103.4 (14)
W3A—O13A—W8A	124.3 (6)	N23S—C24S—H24A	111.1
W9A—O14A—W3A	126.7 (6)	C25S—C24S—H24A	111.1
P1A—O15A—W3A	126.5 (6)	N23S—C24S—H24B	111.1
P1A—O15A—W8A	124.8 (7)	C25S—C24S—H24B	111.1
W3A—O15A—W8A	90.2 (4)	H24A—C24S—H24B	109.1
P1A—O15A—W9A	124.6 (6)	O22S—C25S—N21S	128 (2)
W3A—O15A—W9A	90.1 (4)	O22S—C25S—C24S	126.3 (17)
W8A—O15A—W9A	89.5 (3)	N21S—C25S—C24S	105.6 (17)
W4A—O17A—W5A	124.8 (6)	C35S—N31S—C32S	111.1 (15)
W9A—O18A—W4A	150.6 (7)	C35S—N31S—H31A	124.4
W4A—O19A—W10A	154.2 (7)	C32S—N31S—H31A	124.4
W5A—O21A—W6A	151.0 (7)	O31S—C32S—N31S	123.5 (16)
W5A—O22A—W11A	151.1 (7)	O31S—C32S—N33S	130.1 (17)
W7A—O24A—W6A	127.8 (7)	N31S—C32S—N33S	106.3 (14)
W6A—O25A—W11A	152.6 (7)	C32S—N33S—C34S	109.5 (17)
W7A—O27A—W8A	150.2 (7)	C32S—N33S—H33A	125.3
W12A—O28A—W7A	152.7 (7)	C34S—N33S—H33A	125.3
W8A—O30A—W9A	125.0 (5)	C35S—C34S—N33S	104.1 (17)
W8A—O31A—W12A	153.0 (6)	C35S—C34S—H34A	110.9
W9A—O33A—W10A	152.7 (6)	N33S—C34S—H34A	110.9
W10A—O35A—W11A	127.0 (7)	C35S—C34S—H34B	110.9
W10A—O36A—W12A	126.1 (6)	N33S—C34S—H34B	110.9
P1A—O37A—W11A	126.3 (6)	H34A—C34S—H34B	109.0
P1A—O37A—W10A	127.0 (7)	O32S—C35S—N31S	127.0 (17)
W11A—O37A—W10A	89.3 (4)	O32S—C35S—C34S	124.1 (17)
P1A—O37A—W12A	124.5 (7)	N31S—C35S—C34S	108.9 (16)
W11A—O37A—W12A	89.0 (4)	C45S—N41S—C42S	109.8 (19)
W10A—O37A—W12A	88.9 (4)	C45S—N41S—H41A	125.1
W12A—O39A—W11A	125.6 (7)	C42S—N41S—H41A	125.1
O1B—W1B—O3B	104.5 (5)	O41S—C42S—N41S	124 (2)
O1B—W1B—O2B	104.7 (6)	O41S—C42S—N43S	126 (2)
O3B—W1B—O2B	85.3 (5)	N41S—C42S—N43S	109.4 (18)
O1B—W1B—O4B	101.4 (5)	C42S—N43S—C44S	104.6 (17)
O3B—W1B—O4B	89.8 (5)	C42S—N43S—H43A	127.7
O2B—W1B—O4B	153.9 (5)	C44S—N43S—H43A	127.7
O1B—W1B—O5B	102.0 (5)	C45S—C44S—N43S	107.7 (16)
O3B—W1B—O5B	153.5 (5)	C45S—C44S—H44A	110.2
O2B—W1B—O5B	87.5 (5)	N43S—C44S—H44A	110.2
O4B—W1B—O5B	85.6 (5)	C45S—C44S—H44B	110.2
O1B—W1B—O6B	170.5 (5)	N43S—C44S—H44B	110.2
O3B—W1B—O6B	82.5 (4)	H44A—C44S—H44B	108.5
O2B—W1B—O6B	82.0 (4)	O42S—C45S—N41S	125 (2)
O4B—W1B—O6B	71.9 (4)	O42S—C45S—C44S	127 (2)
O5B—W1B—O6B	71.2 (4)	N41S—C45S—C44S	108 (2)
O7B—W2B—O9B	103.9 (6)	C55S—N51S—C52S	112.3 (15)
O7B—W2B—O2B	104.3 (5)	C55S—N51S—H51A	123.8
O9B—W2B—O2B	91.1 (5)	C52S—N51S—H51A	123.8
O7B—W2B—O10B	101.0 (6)	O51S—C52S—N53S	127.1 (18)
O9B—W2B—O10B	86.3 (6)	O51S—C52S—N51S	126.2 (18)
O2B—W2B—O10B	154.4 (5)	N53S—C52S—N51S	106.5 (15)
O7B—W2B—O8B	101.8 (5)	C52S—N53S—C54S	111.7 (14)
O9B—W2B—O8B	154.3 (5)	C52S—N53S—C71S	121.7 (15)
O2B—W2B—O8B	84.2 (5)	C54S—N53S—C71S	126.1 (15)
O10B—W2B—O8B	87.1 (5)	N53S—C54S—C55S	102.9 (14)
O7B—W2B—O11B	171.4 (5)	N53S—C54S—H54A	111.2
O9B—W2B—O11B	72.3 (5)	C55S—C54S—H54A	111.2
O2B—W2B—O11B	83.6 (4)	N53S—C54S—H54B	111.2
O10B—W2B—O11B	71.3 (5)	C55S—C54S—H54B	111.2
O8B—W2B—O11B	82.1 (4)	H54A—C54S—H54B	109.1
O12B—W3B—O8B	103.1 (6)	O52S—C55S—N51S	126.4 (16)
O12B—W3B—O13B	102.2 (5)	O52S—C55S—C54S	127.3 (17)
O8B—W3B—O13B	89.9 (5)	N51S—C55S—C54S	106.3 (14)
O12B—W3B—O3B	102.8 (5)	C65S—N61S—C62S	111 (2)
O8B—W3B—O3B	85.7 (5)	C65S—N61S—H61A	124.3
O13B—W3B—O3B	155.0 (5)	C62S—N61S—H61A	124.3
O12B—W3B—O14B	103.1 (6)	O61S—C62S—N63S	131 (3)
O8B—W3B—O14B	153.7 (5)	O61S—C62S—N61S	124 (3)
O13B—W3B—O14B	87.0 (5)	N63S—C62S—N61S	105 (2)
O3B—W3B—O14B	86.1 (5)	C62S—N63S—C64S	113.7 (18)
O12B—W3B—O15B	172.6 (5)	C62S—N63S—C71S	121 (2)
O8B—W3B—O15B	82.7 (4)	C64S—N63S—C71S	123.1 (16)
O13B—W3B—O15B	73.0 (4)	N63S—C64S—C65S	101.2 (15)
O3B—W3B—O15B	82.0 (4)	N63S—C64S—H64A	111.5
O14B—W3B—O15B	71.4 (4)	C65S—C64S—H64A	111.5
O16B—W4B—O4B	102.0 (5)	N63S—C64S—H64B	111.5
O16B—W4B—O19B	101.1 (6)	C65S—C64S—H64B	111.5
O4B—W4B—O19B	156.9 (5)	H64A—C64S—H64B	109.4
O16B—W4B—O17B	100.3 (5)	O62S—C65S—N61S	129 (2)
O4B—W4B—O17B	88.5 (5)	O62S—C65S—C64S	123 (2)
O19B—W4B—O17B	87.9 (5)	N61S—C65S—C64S	108.4 (17)
O16B—W4B—O18B	103.0 (5)	N63S—C71S—N53S	115.7 (15)
O4B—W4B—O18B	88.4 (5)	N63S—C71S—H71A	108.3
O19B—W4B—O18B	86.0 (5)	N53S—C71S—H71A	108.4
O17B—W4B—O18B	156.7 (5)	N63S—C71S—H71B	108.4
O16B—W4B—O6B	172.0 (5)	N53S—C71S—H71B	108.3
O4B—W4B—O6B	73.3 (4)	H71A—C71S—H71B	107.4
			
O7A—W2A—O2A—W1A	−126.1 (16)	O17B—W5B—O5B—W1B	71.1 (8)
O9A—W2A—O2A—W1A	129.1 (16)	O22B—W5B—O5B—W1B	−1.9 (17)
O8A—W2A—O2A—W1A	−26.0 (16)	O6B—W5B—O5B—W1B	−1.2 (6)
O10A—W2A—O2A—W1A	45 (3)	O1B—W1B—O5B—W5B	−171.9 (7)
O11A—W2A—O2A—W1A	57.3 (16)	O3B—W1B—O5B—W5B	9.4 (16)
O1A—W1A—O2A—W2A	127.7 (16)	O2B—W1B—O5B—W5B	83.7 (8)
O4A—W1A—O2A—W2A	−54 (2)	O4B—W1B—O5B—W5B	−71.2 (8)
O3A—W1A—O2A—W2A	26.5 (16)	O6B—W1B—O5B—W5B	1.2 (6)
O5A—W1A—O2A—W2A	−129.0 (16)	O15B—P1B—O6B—W4B	−60.5 (10)
O6A—W1A—O2A—W2A	−57.5 (16)	O11B—P1B—O6B—W4B	178.0 (7)
O12A—W3A—O3A—W1A	130.6 (14)	O37B—P1B—O6B—W4B	58.5 (10)
O8A—W3A—O3A—W1A	28.0 (14)	O15B—P1B—O6B—W5B	178.6 (7)
O13A—W3A—O3A—W1A	−52 (2)	O11B—P1B—O6B—W5B	57.1 (10)
O14A—W3A—O3A—W1A	−127.7 (15)	O37B—P1B—O6B—W5B	−62.4 (9)
O15A—W3A—O3A—W1A	−56.2 (14)	O15B—P1B—O6B—W1B	60.3 (9)
O1A—W1A—O3A—W3A	−130.3 (14)	O11B—P1B—O6B—W1B	−61.2 (9)
O4A—W1A—O3A—W3A	126.8 (15)	O37B—P1B—O6B—W1B	179.3 (7)
O5A—W1A—O3A—W3A	47 (2)	O16B—W4B—O6B—P1B	−171 (3)
O2A—W1A—O3A—W3A	−27.5 (14)	O4B—W4B—O6B—P1B	134.8 (9)
O6A—W1A—O3A—W3A	54.9 (14)	O19B—W4B—O6B—P1B	−42.1 (9)
O1A—W1A—O4A—W4A	170.9 (8)	O17B—W4B—O6B—P1B	−131.7 (9)
O3A—W1A—O4A—W4A	−87.1 (8)	O18B—W4B—O6B—P1B	44.5 (8)
O5A—W1A—O4A—W4A	67.9 (8)	O16B—W4B—O6B—W5B	−35 (3)
O2A—W1A—O4A—W4A	−7.4 (17)	O4B—W4B—O6B—W5B	−89.8 (5)
O6A—W1A—O4A—W4A	−3.7 (7)	O19B—W4B—O6B—W5B	93.3 (4)
O16A—W4A—O4A—W1A	−170.7 (8)	O17B—W4B—O6B—W5B	3.7 (4)
O19A—W4A—O4A—W1A	16.5 (17)	O18B—W4B—O6B—W5B	179.9 (5)
O17A—W4A—O4A—W1A	−69.8 (8)	O16B—W4B—O6B—W1B	54 (3)
O18A—W4A—O4A—W1A	86.9 (8)	O4B—W4B—O6B—W1B	−1.1 (4)
O6A—W4A—O4A—W1A	3.7 (7)	O19B—W4B—O6B—W1B	−178.0 (4)
O20A—W5A—O5A—W1A	172.2 (8)	O17B—W4B—O6B—W1B	92.4 (5)
O21A—W5A—O5A—W1A	−84.1 (8)	O18B—W4B—O6B—W1B	−91.3 (5)
O22A—W5A—O5A—W1A	1.4 (16)	O20B—W5B—O6B—P1B	174 (4)
O17A—W5A—O5A—W1A	72.6 (8)	O5B—W5B—O6B—P1B	−133.6 (9)
O6A—W5A—O5A—W1A	−1.0 (6)	O21B—W5B—O6B—P1B	−40.2 (8)
O1A—W1A—O5A—W5A	−173.5 (7)	O17B—W5B—O6B—P1B	132.9 (9)
O4A—W1A—O5A—W5A	−71.3 (8)	O22B—W5B—O6B—P1B	46.1 (8)
O3A—W1A—O5A—W5A	9.2 (16)	O20B—W5B—O6B—W4B	38 (4)
O2A—W1A—O5A—W5A	83.5 (8)	O5B—W5B—O6B—W4B	89.9 (5)
O6A—W1A—O5A—W5A	1.0 (6)	O21B—W5B—O6B—W4B	−176.7 (5)
O37A—P1A—O6A—W5A	−61.9 (9)	O17B—W5B—O6B—W4B	−3.6 (4)
O11A—P1A—O6A—W5A	58.8 (9)	O22B—W5B—O6B—W4B	−90.4 (4)
O15A—P1A—O6A—W5A	177.6 (7)	O20B—W5B—O6B—W1B	−51 (4)
O37A—P1A—O6A—W4A	58.5 (10)	O5B—W5B—O6B—W1B	0.8 (4)
O11A—P1A—O6A—W4A	179.1 (7)	O21B—W5B—O6B—W1B	94.2 (4)
O15A—P1A—O6A—W4A	−62.1 (9)	O17B—W5B—O6B—W1B	−92.7 (4)
O37A—P1A—O6A—W1A	178.6 (7)	O22B—W5B—O6B—W1B	−179.5 (4)
O11A—P1A—O6A—W1A	−60.8 (9)	O1B—W1B—O6B—P1B	179 (3)
O15A—P1A—O6A—W1A	58.0 (9)	O3B—W1B—O6B—P1B	−43.2 (8)
O20A—W5A—O6A—P1A	176 (3)	O2B—W1B—O6B—P1B	43.1 (8)
O5A—W5A—O6A—P1A	−135.2 (9)	O4B—W1B—O6B—P1B	−135.4 (9)
O21A—W5A—O6A—P1A	−42.5 (8)	O5B—W1B—O6B—P1B	133.1 (9)
O22A—W5A—O6A—P1A	45.9 (8)	O1B—W1B—O6B—W4B	−45 (3)
O17A—W5A—O6A—P1A	134.7 (9)	O3B—W1B—O6B—W4B	93.3 (5)
O20A—W5A—O6A—W4A	40 (3)	O2B—W1B—O6B—W4B	179.6 (5)
O5A—W5A—O6A—W4A	88.8 (4)	O4B—W1B—O6B—W4B	1.1 (4)
O21A—W5A—O6A—W4A	−178.5 (5)	O5B—W1B—O6B—W4B	−90.4 (4)
O22A—W5A—O6A—W4A	−90.2 (5)	O1B—W1B—O6B—W5B	45 (3)
O17A—W5A—O6A—W4A	−1.3 (4)	O3B—W1B—O6B—W5B	−177.1 (5)
O20A—W5A—O6A—W1A	−48 (3)	O2B—W1B—O6B—W5B	−90.8 (4)
O5A—W5A—O6A—W1A	0.6 (4)	O4B—W1B—O6B—W5B	90.7 (5)
O21A—W5A—O6A—W1A	93.3 (4)	O5B—W1B—O6B—W5B	−0.8 (4)
O22A—W5A—O6A—W1A	−178.3 (5)	O12B—W3B—O8B—W2B	−124.8 (14)
O17A—W5A—O6A—W1A	−89.5 (5)	O13B—W3B—O8B—W2B	132.7 (15)
O16A—W4A—O6A—P1A	−180 (88)	O3B—W3B—O8B—W2B	−22.6 (14)
O19A—W4A—O6A—P1A	−40.7 (8)	O14B—W3B—O8B—W2B	50 (2)
O17A—W4A—O6A—P1A	−134.2 (9)	O15B—W3B—O8B—W2B	59.8 (14)
O4A—W4A—O6A—P1A	133.8 (9)	O7B—W2B—O8B—W3B	126.6 (14)
O18A—W4A—O6A—P1A	45.1 (9)	O9B—W2B—O8B—W3B	−57 (2)
O16A—W4A—O6A—W5A	−44 (4)	O2B—W2B—O8B—W3B	23.1 (14)
O19A—W4A—O6A—W5A	94.8 (5)	O10B—W2B—O8B—W3B	−132.8 (15)
O17A—W4A—O6A—W5A	1.3 (4)	O11B—W2B—O8B—W3B	−61.2 (14)
O4A—W4A—O6A—W5A	−90.7 (5)	O7B—W2B—O9B—W6B	169.0 (8)
O18A—W4A—O6A—W5A	−179.5 (5)	O2B—W2B—O9B—W6B	−86.0 (8)
O16A—W4A—O6A—W1A	44 (4)	O10B—W2B—O9B—W6B	68.5 (8)
O19A—W4A—O6A—W1A	−176.8 (5)	O8B—W2B—O9B—W6B	−7.1 (17)
O17A—W4A—O6A—W1A	89.7 (5)	O11B—W2B—O9B—W6B	−3.0 (7)
O4A—W4A—O6A—W1A	−2.3 (4)	O23B—W6B—O9B—W2B	−172.0 (8)
O18A—W4A—O6A—W1A	−91.0 (4)	O24B—W6B—O9B—W2B	−69.5 (8)
O1A—W1A—O6A—P1A	−179 (3)	O25B—W6B—O9B—W2B	17.4 (17)
O4A—W1A—O6A—P1A	−133.6 (9)	O21B—W6B—O9B—W2B	86.8 (8)
O3A—W1A—O6A—P1A	−42.1 (8)	O11B—W6B—O9B—W2B	3.0 (7)
O5A—W1A—O6A—P1A	134.4 (8)	O26B—W7B—O10B—W2B	171.5 (8)
O2A—W1A—O6A—P1A	44.8 (8)	O27B—W7B—O10B—W2B	−84.7 (9)
O1A—W1A—O6A—W5A	46 (4)	O28B—W7B—O10B—W2B	−3.0 (17)
O4A—W1A—O6A—W5A	91.4 (4)	O24B—W7B—O10B—W2B	69.6 (9)
O3A—W1A—O6A—W5A	−177.1 (4)	O11B—W7B—O10B—W2B	−1.9 (7)
O5A—W1A—O6A—W5A	−0.6 (4)	O7B—W2B—O10B—W7B	−174.1 (8)
O2A—W1A—O6A—W5A	−90.2 (4)	O9B—W2B—O10B—W7B	−70.7 (9)
O1A—W1A—O6A—W4A	−43 (4)	O2B—W2B—O10B—W7B	14.3 (18)
O4A—W1A—O6A—W4A	2.3 (4)	O8B—W2B—O10B—W7B	84.5 (9)
O3A—W1A—O6A—W4A	93.8 (4)	O11B—W2B—O10B—W7B	1.8 (7)
O5A—W1A—O6A—W4A	−89.7 (4)	O15B—P1B—O11B—W6B	179.6 (7)
O2A—W1A—O6A—W4A	−179.3 (4)	O6B—P1B—O11B—W6B	−60.0 (9)
O12A—W3A—O8A—W2A	−130.7 (14)	O37B—P1B—O11B—W6B	59.5 (9)
O3A—W3A—O8A—W2A	−28.4 (15)	O15B—P1B—O11B—W7B	57.0 (9)
O13A—W3A—O8A—W2A	129.2 (15)	O6B—P1B—O11B—W7B	177.5 (7)
O14A—W3A—O8A—W2A	46 (2)	O37B—P1B—O11B—W7B	−63.0 (9)
O15A—W3A—O8A—W2A	56.1 (14)	O15B—P1B—O11B—W2B	−61.9 (9)
O7A—W2A—O8A—W3A	128.4 (15)	O6B—P1B—O11B—W2B	58.6 (9)
O9A—W2A—O8A—W3A	−51 (2)	O37B—P1B—O11B—W2B	178.1 (7)
O2A—W2A—O8A—W3A	27.8 (15)	O23B—W6B—O11B—P1B	177 (4)
O10A—W2A—O8A—W3A	−128.9 (15)	O24B—W6B—O11B—P1B	−135.8 (9)
O11A—W2A—O8A—W3A	−56.8 (14)	O25B—W6B—O11B—P1B	−42.3 (8)
O7A—W2A—O9A—W6A	170.9 (8)	O21B—W6B—O11B—P1B	43.3 (8)
O2A—W2A—O9A—W6A	−87.4 (8)	O9B—W6B—O11B—P1B	131.8 (9)
O8A—W2A—O9A—W6A	−9.4 (17)	O23B—W6B—O11B—W7B	−45 (4)
O10A—W2A—O9A—W6A	68.0 (8)	O24B—W6B—O11B—W7B	1.8 (4)
O11A—W2A—O9A—W6A	−3.6 (7)	O25B—W6B—O11B—W7B	95.3 (4)
O23A—W6A—O9A—W2A	−171.8 (8)	O21B—W6B—O11B—W7B	−179.1 (4)
O24A—W6A—O9A—W2A	−67.5 (8)	O9B—W6B—O11B—W7B	−90.6 (5)
O25A—W6A—O9A—W2A	17.6 (16)	O23B—W6B—O11B—W2B	43 (4)
O21A—W6A—O9A—W2A	86.2 (8)	O24B—W6B—O11B—W2B	90.6 (5)
O11A—W6A—O9A—W2A	3.6 (7)	O25B—W6B—O11B—W2B	−175.9 (4)
O26A—W7A—O10A—W2A	169.2 (8)	O21B—W6B—O11B—W2B	−90.3 (4)
O24A—W7A—O10A—W2A	67.3 (8)	O9B—W6B—O11B—W2B	−1.9 (4)
O27A—W7A—O10A—W2A	−88.7 (8)	O26B—W7B—O11B—P1B	175 (3)
O28A—W7A—O10A—W2A	−12.8 (17)	O27B—W7B—O11B—P1B	−40.5 (8)
O11A—W7A—O10A—W2A	−4.5 (7)	O28B—W7B—O11B—P1B	46.8 (8)
O7A—W2A—O10A—W7A	−171.5 (8)	O10B—W7B—O11B—P1B	−132.6 (9)
O9A—W2A—O10A—W7A	−67.2 (8)	O24B—W7B—O11B—P1B	135.8 (9)
O2A—W2A—O10A—W7A	17.8 (18)	O26B—W7B—O11B—W6B	38 (4)
O8A—W2A—O10A—W7A	88.1 (8)	O27B—W7B—O11B—W6B	−178.1 (5)
O11A—W2A—O10A—W7A	4.5 (7)	O28B—W7B—O11B—W6B	−90.7 (4)
O6A—P1A—O11A—W2A	58.7 (9)	O10B—W7B—O11B—W6B	89.8 (5)
O37A—P1A—O11A—W2A	178.1 (7)	O24B—W7B—O11B—W6B	−1.8 (4)
O15A—P1A—O11A—W2A	−59.9 (9)	O26B—W7B—O11B—W2B	−51 (4)
O6A—P1A—O11A—W7A	179.4 (7)	O27B—W7B—O11B—W2B	93.3 (5)
O37A—P1A—O11A—W7A	−61.2 (9)	O28B—W7B—O11B—W2B	−179.4 (4)
O15A—P1A—O11A—W7A	60.7 (9)	O10B—W7B—O11B—W2B	1.1 (4)
O6A—P1A—O11A—W6A	−62.3 (9)	O24B—W7B—O11B—W2B	−90.4 (5)
O37A—P1A—O11A—W6A	57.1 (9)	O7B—W2B—O11B—P1B	162 (3)
O15A—P1A—O11A—W6A	179.1 (7)	O9B—W2B—O11B—P1B	−133.4 (9)
O7A—W2A—O11A—P1A	170 (4)	O2B—W2B—O11B—P1B	−40.2 (8)
O9A—W2A—O11A—P1A	−133.9 (8)	O10B—W2B—O11B—P1B	134.4 (9)
O2A—W2A—O11A—P1A	−42.0 (8)	O8B—W2B—O11B—P1B	44.8 (8)
O8A—W2A—O11A—P1A	43.6 (8)	O7B—W2B—O11B—W6B	−62 (3)
O10A—W2A—O11A—P1A	132.4 (9)	O9B—W2B—O11B—W6B	1.9 (4)
O7A—W2A—O11A—W7A	35 (5)	O2B—W2B—O11B—W6B	95.2 (4)
O9A—W2A—O11A—W7A	90.8 (5)	O10B—W2B—O11B—W6B	−90.2 (5)
O2A—W2A—O11A—W7A	−177.3 (4)	O8B—W2B—O11B—W6B	−179.9 (4)
O8A—W2A—O11A—W7A	−91.7 (4)	O7B—W2B—O11B—W7B	27 (3)
O10A—W2A—O11A—W7A	−2.9 (4)	O9B—W2B—O11B—W7B	91.0 (5)
O7A—W2A—O11A—W6A	−54 (4)	O2B—W2B—O11B—W7B	−175.7 (4)
O9A—W2A—O11A—W6A	2.2 (4)	O10B—W2B—O11B—W7B	−1.1 (4)
O2A—W2A—O11A—W6A	94.1 (4)	O8B—W2B—O11B—W7B	−90.8 (4)
O8A—W2A—O11A—W6A	179.7 (4)	O29B—W8B—O13B—W3B	−173.4 (7)
O10A—W2A—O11A—W6A	−91.4 (5)	O31B—W8B—O13B—W3B	9.2 (16)
O26A—W7A—O11A—P1A	174 (4)	O27B—W8B—O13B—W3B	84.2 (7)
O24A—W7A—O11A—P1A	132.8 (9)	O30B—W8B—O13B—W3B	−69.8 (8)
O10A—W7A—O11A—P1A	−133.0 (9)	O15B—W8B—O13B—W3B	1.4 (6)
O27A—W7A—O11A—P1A	−43.7 (8)	O12B—W3B—O13B—W8B	172.8 (8)
O28A—W7A—O11A—P1A	43.5 (8)	O8B—W3B—O13B—W8B	−83.7 (8)
O26A—W7A—O11A—W2A	−50 (4)	O3B—W3B—O13B—W8B	−4.1 (17)
O24A—W7A—O11A—W2A	−91.3 (4)	O14B—W3B—O13B—W8B	70.1 (8)
O10A—W7A—O11A—W2A	2.9 (4)	O15B—W3B—O13B—W8B	−1.3 (6)
O27A—W7A—O11A—W2A	92.3 (4)	O32B—W9B—O14B—W3B	169.7 (7)
O28A—W7A—O11A—W2A	179.4 (4)	O30B—W9B—O14B—W3B	67.0 (8)
O26A—W7A—O11A—W6A	39 (4)	O18B—W9B—O14B—W3B	−88.6 (8)
O24A—W7A—O11A—W6A	−1.8 (4)	O33B—W9B—O14B—W3B	−12.3 (17)
O10A—W7A—O11A—W6A	92.4 (5)	O15B—W9B—O14B—W3B	−4.8 (6)
O27A—W7A—O11A—W6A	−178.3 (4)	O12B—W3B—O14B—W9B	−170.2 (7)
O28A—W7A—O11A—W6A	−91.2 (4)	O8B—W3B—O14B—W9B	15.4 (16)
O23A—W6A—O11A—P1A	165 (3)	O13B—W3B—O14B—W9B	−68.3 (8)
O24A—W6A—O11A—P1A	−132.1 (8)	O3B—W3B—O14B—W9B	87.6 (8)
O25A—W6A—O11A—P1A	−39.9 (8)	O15B—W3B—O14B—W9B	4.8 (6)
O9A—W6A—O11A—P1A	133.9 (9)	O11B—P1B—O15B—W9B	−179.7 (7)
O21A—W6A—O11A—P1A	46.6 (8)	O6B—P1B—O15B—W9B	58.7 (10)
O23A—W6A—O11A—W2A	29 (3)	O37B—P1B—O15B—W9B	−60.0 (9)
O24A—W6A—O11A—W2A	91.8 (5)	O11B—P1B—O15B—W8B	−58.6 (10)
O25A—W6A—O11A—W2A	−176.1 (5)	O6B—P1B—O15B—W8B	179.8 (7)
O9A—W6A—O11A—W2A	−2.2 (4)	O37B—P1B—O15B—W8B	61.1 (10)
O21A—W6A—O11A—W2A	−89.6 (5)	O11B—P1B—O15B—W3B	59.7 (9)
O23A—W6A—O11A—W7A	−61 (3)	O6B—P1B—O15B—W3B	−61.9 (9)
O24A—W6A—O11A—W7A	1.8 (4)	O37B—P1B—O15B—W3B	179.5 (7)
O25A—W6A—O11A—W7A	94.0 (4)	O32B—W9B—O15B—P1B	−177 (3)
O9A—W6A—O11A—W7A	−92.1 (5)	O14B—W9B—O15B—P1B	−132.5 (9)
O21A—W6A—O11A—W7A	−179.5 (5)	O30B—W9B—O15B—P1B	132.7 (9)
O12A—W3A—O13A—W8A	170.4 (7)	O18B—W9B—O15B—P1B	−42.0 (9)
O8A—W3A—O13A—W8A	−86.2 (7)	O33B—W9B—O15B—P1B	44.3 (8)
O3A—W3A—O13A—W8A	−6.9 (16)	O32B—W9B—O15B—W8B	47 (4)
O14A—W3A—O13A—W8A	68.7 (7)	O14B—W9B—O15B—W8B	91.4 (5)
O15A—W3A—O13A—W8A	−2.7 (6)	O30B—W9B—O15B—W8B	−3.5 (5)
O29A—W8A—O13A—W3A	−172.3 (7)	O18B—W9B—O15B—W8B	−178.1 (5)
O31A—W8A—O13A—W3A	13.4 (15)	O33B—W9B—O15B—W8B	−91.8 (5)
O30A—W8A—O13A—W3A	−70.3 (7)	O32B—W9B—O15B—W3B	−42 (4)
O27A—W8A—O13A—W3A	86.2 (7)	O14B—W9B—O15B—W3B	3.1 (4)
O15A—W8A—O13A—W3A	2.7 (6)	O30B—W9B—O15B—W3B	−91.8 (5)
O32A—W9A—O14A—W3A	170.8 (8)	O18B—W9B—O15B—W3B	93.6 (5)
O18A—W9A—O14A—W3A	−86.4 (8)	O33B—W9B—O15B—W3B	179.9 (5)
O33A—W9A—O14A—W3A	−8.3 (16)	O29B—W8B—O15B—P1B	179 (88)
O30A—W9A—O14A—W3A	70.3 (7)	O13B—W8B—O15B—P1B	133.5 (9)
O15A—W9A—O14A—W3A	−2.2 (6)	O31B—W8B—O15B—P1B	−43.3 (9)
O12A—W3A—O14A—W9A	−170.5 (8)	O27B—W8B—O15B—P1B	43.2 (9)
O8A—W3A—O14A—W9A	12.6 (16)	O30B—W8B—O15B—P1B	−133.6 (10)
O3A—W3A—O14A—W9A	87.0 (8)	O29B—W8B—O15B—W9B	−44 (4)
O13A—W3A—O14A—W9A	−71.0 (7)	O13B—W8B—O15B—W9B	−89.6 (5)
O15A—W3A—O14A—W9A	2.2 (6)	O31B—W8B—O15B—W9B	93.7 (4)
O6A—P1A—O15A—W3A	−60.1 (9)	O27B—W8B—O15B—W9B	−179.8 (5)
O37A—P1A—O15A—W3A	−179.4 (7)	O30B—W8B—O15B—W9B	3.4 (5)
O11A—P1A—O15A—W3A	58.7 (9)	O29B—W8B—O15B—W3B	45 (4)
O6A—P1A—O15A—W8A	178.6 (7)	O13B—W8B—O15B—W3B	−0.9 (4)
O37A—P1A—O15A—W8A	59.2 (9)	O31B—W8B—O15B—W3B	−177.6 (4)
O11A—P1A—O15A—W8A	−62.7 (9)	O27B—W8B—O15B—W3B	−91.1 (5)
O6A—P1A—O15A—W9A	60.8 (9)	O30B—W8B—O15B—W3B	92.1 (5)
O37A—P1A—O15A—W9A	−58.5 (10)	O12B—W3B—O15B—P1B	176 (4)
O11A—P1A—O15A—W9A	179.6 (7)	O8B—W3B—O15B—P1B	−41.6 (8)
O12A—W3A—O15A—P1A	−178 (3)	O13B—W3B—O15B—P1B	−133.7 (9)
O8A—W3A—O15A—P1A	−41.9 (8)	O3B—W3B—O15B—P1B	45.1 (8)
O3A—W3A—O15A—P1A	44.7 (8)	O14B—W3B—O15B—P1B	133.7 (9)
O13A—W3A—O15A—P1A	−133.7 (9)	O12B—W3B—O15B—W9B	39 (4)
O14A—W3A—O15A—P1A	133.7 (9)	O8B—W3B—O15B—W9B	−178.2 (4)
O12A—W3A—O15A—W8A	−43 (3)	O13B—W3B—O15B—W9B	89.6 (5)
O8A—W3A—O15A—W8A	93.6 (4)	O3B—W3B—O15B—W9B	−91.6 (4)
O3A—W3A—O15A—W8A	−179.8 (4)	O14B—W3B—O15B—W9B	−3.0 (4)
O13A—W3A—O15A—W8A	1.8 (4)	O12B—W3B—O15B—W8B	−50 (4)
O14A—W3A—O15A—W8A	−90.8 (4)	O8B—W3B—O15B—W8B	93.0 (4)
O12A—W3A—O15A—W9A	47 (3)	O13B—W3B—O15B—W8B	0.9 (4)
O8A—W3A—O15A—W9A	−176.9 (5)	O3B—W3B—O15B—W8B	179.7 (5)
O3A—W3A—O15A—W9A	−90.4 (4)	O14B—W3B—O15B—W8B	−91.7 (5)
O13A—W3A—O15A—W9A	91.2 (4)	O16B—W4B—O17B—W5B	169.4 (7)
O14A—W3A—O15A—W9A	−1.4 (4)	O4B—W4B—O17B—W5B	67.5 (7)
O29A—W8A—O15A—P1A	170 (3)	O19B—W4B—O17B—W5B	−89.7 (7)
O31A—W8A—O15A—P1A	−40.7 (8)	O18B—W4B—O17B—W5B	−15.0 (17)
O30A—W8A—O15A—P1A	−132.1 (8)	O6B—W4B—O17B—W5B	−5.6 (6)
O27A—W8A—O15A—P1A	46.3 (8)	O20B—W5B—O17B—W4B	−169.4 (7)
O13A—W8A—O15A—P1A	134.9 (8)	O5B—W5B—O17B—W4B	−67.4 (7)
O29A—W8A—O15A—W3A	34 (3)	O21B—W5B—O17B—W4B	23.0 (16)
O31A—W8A—O15A—W3A	−177.3 (4)	O22B—W5B—O17B—W4B	90.1 (7)
O30A—W8A—O15A—W3A	91.3 (5)	O6B—W5B—O17B—W4B	5.5 (6)
O27A—W8A—O15A—W3A	−90.3 (4)	O32B—W9B—O18B—W4B	−132.3 (14)
O13A—W8A—O15A—W3A	−1.8 (4)	O14B—W9B—O18B—W4B	126.6 (14)
O29A—W8A—O15A—W9A	−56 (3)	O30B—W9B—O18B—W4B	41 (2)
O31A—W8A—O15A—W9A	92.6 (4)	O33B—W9B—O18B—W4B	−29.2 (14)
O30A—W8A—O15A—W9A	1.2 (4)	O15B—W9B—O18B—W4B	53.3 (14)
O27A—W8A—O15A—W9A	179.6 (5)	O16B—W4B—O18B—W9B	130.3 (14)
O13A—W8A—O15A—W9A	−91.9 (4)	O4B—W4B—O18B—W9B	−127.8 (14)
O32A—W9A—O15A—P1A	−179 (54)	O19B—W4B—O18B—W9B	29.8 (14)
O14A—W9A—O15A—P1A	−135.0 (9)	O17B—W4B—O18B—W9B	−45 (2)
O18A—W9A—O15A—P1A	−44.4 (8)	O6B—W4B—O18B—W9B	−54.4 (14)
O33A—W9A—O15A—P1A	42.5 (8)	O34B—W10B—O19B—W4B	126.8 (14)
O30A—W9A—O15A—P1A	132.2 (9)	O33B—W10B—O19B—W4B	25.0 (14)
O32A—W9A—O15A—W3A	−43 (3)	O36B—W10B—O19B—W4B	−59 (2)
O14A—W9A—O15A—W3A	1.4 (4)	O35B—W10B—O19B—W4B	−131.6 (15)
O18A—W9A—O15A—W3A	92.1 (5)	O37B—W10B—O19B—W4B	−59.0 (14)
O33A—W9A—O15A—W3A	178.9 (4)	O16B—W4B—O19B—W10B	−128.7 (14)
O30A—W9A—O15A—W3A	−91.3 (5)	O4B—W4B—O19B—W10B	50 (2)
O32A—W9A—O15A—W8A	48 (3)	O17B—W4B—O19B—W10B	131.3 (15)
O14A—W9A—O15A—W8A	91.6 (5)	O18B—W4B—O19B—W10B	−26.2 (14)
O18A—W9A—O15A—W8A	−177.7 (5)	O6B—W4B—O19B—W10B	57.7 (14)
O33A—W9A—O15A—W8A	−90.9 (4)	O20B—W5B—O21B—W6B	−130.0 (14)
O30A—W9A—O15A—W8A	−1.1 (4)	O5B—W5B—O21B—W6B	127.4 (14)
O16A—W4A—O17A—W5A	172.5 (8)	O17B—W5B—O21B—W6B	38 (2)
O19A—W4A—O17A—W5A	−84.4 (8)	O22B—W5B—O21B—W6B	−29.3 (14)
O4A—W4A—O17A—W5A	70.4 (8)	O6B—W5B—O21B—W6B	54.4 (14)
O18A—W4A—O17A—W5A	−3.9 (18)	O23B—W6B—O21B—W5B	130.2 (14)
O6A—W4A—O17A—W5A	−2.0 (7)	O24B—W6B—O21B—W5B	−53 (2)
O20A—W5A—O17A—W4A	−172.1 (8)	O25B—W6B—O21B—W5B	29.8 (14)
O5A—W5A—O17A—W4A	−71.1 (8)	O9B—W6B—O21B—W5B	−127.2 (15)
O21A—W5A—O17A—W4A	8.9 (18)	O11B—W6B—O21B—W5B	−54.8 (14)
O22A—W5A—O17A—W4A	84.8 (8)	O38B—W11B—O22B—W5B	−131.2 (13)
O6A—W5A—O17A—W4A	2.0 (7)	O35B—W11B—O22B—W5B	128.5 (13)
O32A—W9A—O18A—W4A	−128.7 (13)	O25B—W11B—O22B—W5B	−28.1 (13)
O14A—W9A—O18A—W4A	130.3 (14)	O39B—W11B—O22B—W5B	45 (2)
O33A—W9A—O18A—W4A	−26.2 (13)	O37B—W11B—O22B—W5B	56.0 (13)
O30A—W9A—O18A—W4A	50 (2)	O20B—W5B—O22B—W11B	128.5 (13)
O15A—W9A—O18A—W4A	58.5 (13)	O5B—W5B—O22B—W11B	−57 (2)
O16A—W4A—O18A—W9A	127.4 (14)	O21B—W5B—O22B—W11B	27.9 (13)
O19A—W4A—O18A—W9A	25.6 (14)	O17B—W5B—O22B—W11B	−130.5 (13)
O17A—W4A—O18A—W9A	−56 (2)	O6B—W5B—O22B—W11B	−57.5 (13)
O4A—W4A—O18A—W9A	−130.5 (14)	O23B—W6B—O24B—W7B	172.1 (8)
O6A—W4A—O18A—W9A	−58.0 (13)	O25B—W6B—O24B—W7B	−86.1 (8)
O16A—W4A—O19A—W10A	−129.0 (15)	O21B—W6B—O24B—W7B	−5.0 (17)
O17A—W4A—O19A—W10A	129.1 (15)	O9B—W6B—O24B—W7B	69.3 (8)
O4A—W4A—O19A—W10A	44 (2)	O11B—W6B—O24B—W7B	−2.9 (7)
O18A—W4A—O19A—W10A	−26.8 (15)	O26B—W7B—O24B—W6B	−171.7 (8)
O6A—W4A—O19A—W10A	56.1 (15)	O27B—W7B—O24B—W6B	11.3 (17)
O34A—W10A—O19A—W4A	128.2 (15)	O28B—W7B—O24B—W6B	84.6 (8)
O35A—W10A—O19A—W4A	−129.0 (16)	O10B—W7B—O24B—W6B	−69.6 (8)
O36A—W10A—O19A—W4A	−49 (2)	O11B—W7B—O24B—W6B	2.9 (7)
O33A—W10A—O19A—W4A	26.8 (15)	O23B—W6B—O25B—W11B	−131.5 (15)
O37A—W10A—O19A—W4A	−57.1 (15)	O24B—W6B—O25B—W11B	125.0 (15)
O20A—W5A—O21A—W6A	−125.0 (15)	O21B—W6B—O25B—W11B	−30.5 (15)
O5A—W5A—O21A—W6A	132.8 (15)	O9B—W6B—O25B—W11B	39 (2)
O22A—W5A—O21A—W6A	−21.7 (15)	O11B—W6B—O25B—W11B	52.8 (14)
O17A—W5A—O21A—W6A	54 (2)	O38B—W11B—O25B—W6B	131.7 (14)
O6A—W5A—O21A—W6A	60.6 (15)	O22B—W11B—O25B—W6B	30.7 (15)
O23A—W6A—O21A—W5A	126.2 (15)	O35B—W11B—O25B—W6B	−50 (2)
O24A—W6A—O21A—W5A	−59 (2)	O39B—W11B—O25B—W6B	−126.5 (15)
O25A—W6A—O21A—W5A	22.5 (15)	O37B—W11B—O25B—W6B	−53.5 (14)
O9A—W6A—O21A—W5A	−133.3 (15)	O26B—W7B—O27B—W8B	−127.2 (14)
O11A—W6A—O21A—W5A	−61.8 (15)	O28B—W7B—O27B—W8B	−23.5 (14)
O20A—W5A—O22A—W11A	126.6 (14)	O10B—W7B—O27B—W8B	129.7 (14)
O5A—W5A—O22A—W11A	−63 (2)	O24B—W7B—O27B—W8B	50 (2)
O21A—W5A—O22A—W11A	23.6 (14)	O11B—W7B—O27B—W8B	57.8 (14)
O17A—W5A—O22A—W11A	−133.4 (14)	O29B—W8B—O27B—W7B	126.3 (14)
O6A—W5A—O22A—W11A	−60.4 (13)	O13B—W8B—O27B—W7B	−132.3 (14)
O38A—W11A—O22A—W5A	−126.9 (14)	O31B—W8B—O27B—W7B	24.1 (14)
O25A—W11A—O22A—W5A	−24.9 (14)	O30B—W8B—O27B—W7B	−52 (2)
O35A—W11A—O22A—W5A	131.6 (14)	O15B—W8B—O27B—W7B	−58.8 (14)
O39A—W11A—O22A—W5A	51 (2)	O26B—W7B—O28B—W12B	124.5 (14)
O37A—W11A—O22A—W5A	59.8 (14)	O27B—W7B—O28B—W12B	21.5 (14)
O26A—W7A—O24A—W6A	−171.8 (8)	O10B—W7B—O28B—W12B	−61 (2)
O10A—W7A—O24A—W6A	−69.2 (8)	O24B—W7B—O28B—W12B	−133.6 (14)
O27A—W7A—O24A—W6A	11.5 (17)	O11B—W7B—O28B—W12B	−62.1 (14)
O28A—W7A—O24A—W6A	86.2 (8)	O40B—W12B—O28B—W7B	−126.4 (14)
O11A—W7A—O24A—W6A	3.0 (6)	O31B—W12B—O28B—W7B	−21.7 (14)
O23A—W6A—O24A—W7A	168.9 (7)	O36B—W12B—O28B—W7B	54 (2)
O25A—W6A—O24A—W7A	−86.4 (8)	O39B—W12B—O28B—W7B	134.0 (14)
O9A—W6A—O24A—W7A	67.7 (8)	O37B—W12B—O28B—W7B	61.6 (14)
O21A—W6A—O24A—W7A	−5.8 (16)	O32B—W9B—O30B—W8B	−168.4 (9)
O11A—W6A—O24A—W7A	−3.0 (6)	O14B—W9B—O30B—W8B	−67.1 (9)
O23A—W6A—O25A—W11A	−130.6 (15)	O18B—W9B—O30B—W8B	18.6 (19)
O24A—W6A—O25A—W11A	124.5 (15)	O33B—W9B—O30B—W8B	88.4 (9)
O9A—W6A—O25A—W11A	40 (2)	O15B—W9B—O30B—W8B	5.5 (7)
O21A—W6A—O25A—W11A	−28.5 (15)	O29B—W8B—O30B—W9B	169.0 (9)
O11A—W6A—O25A—W11A	53.2 (15)	O13B—W8B—O30B—W9B	67.8 (9)
O38A—W11A—O25A—W6A	131.7 (15)	O31B—W8B—O30B—W9B	−88.2 (9)
O22A—W11A—O25A—W6A	29.1 (15)	O27B—W8B—O30B—W9B	−12.6 (18)
O35A—W11A—O25A—W6A	−48 (2)	O15B—W8B—O30B—W9B	−5.5 (7)
O39A—W11A—O25A—W6A	−127.0 (15)	O40B—W12B—O31B—W8B	127.5 (13)
O37A—W11A—O25A—W6A	−54.0 (15)	O28B—W12B—O31B—W8B	23.7 (13)
O26A—W7A—O27A—W8A	−125.5 (13)	O36B—W12B—O31B—W8B	−132.8 (14)
O24A—W7A—O27A—W8A	51 (2)	O39B—W12B—O31B—W8B	−58 (2)
O10A—W7A—O27A—W8A	132.2 (13)	O37B—W12B—O31B—W8B	−60.1 (13)
O28A—W7A—O27A—W8A	−23.7 (13)	O29B—W8B—O31B—W12B	−125.9 (13)
O11A—W7A—O27A—W8A	59.3 (13)	O13B—W8B—O31B—W12B	51 (2)
O29A—W8A—O27A—W7A	126.8 (13)	O27B—W8B—O31B—W12B	−24.0 (13)
O31A—W8A—O27A—W7A	23.9 (13)	O30B—W8B—O31B—W12B	130.3 (14)
O30A—W8A—O27A—W7A	−57 (2)	O15B—W8B—O31B—W12B	59.1 (13)
O13A—W8A—O27A—W7A	−132.9 (13)	O34B—W10B—O33B—W9B	−126.9 (15)
O15A—W8A—O27A—W7A	−60.3 (13)	O19B—W10B—O33B—W9B	−25.1 (15)
O40A—W12A—O28A—W7A	−127.4 (14)	O36B—W10B—O33B—W9B	130.3 (15)
O39A—W12A—O28A—W7A	131.9 (14)	O35B—W10B—O33B—W9B	47 (2)
O31A—W12A—O28A—W7A	−23.7 (14)	O37B—W10B—O33B—W9B	57.6 (15)
O36A—W12A—O28A—W7A	50 (2)	O32B—W9B—O33B—W10B	126.9 (15)
O37A—W12A—O28A—W7A	59.4 (13)	O14B—W9B—O33B—W10B	−51 (2)
O26A—W7A—O28A—W12A	126.1 (14)	O30B—W9B—O33B—W10B	−131.1 (15)
O24A—W7A—O28A—W12A	−132.3 (14)	O18B—W9B—O33B—W10B	26.0 (15)
O10A—W7A—O28A—W12A	−52 (2)	O15B—W9B—O33B—W10B	−58.3 (15)
O27A—W7A—O28A—W12A	24.2 (14)	O38B—W11B—O35B—W10B	171.7 (7)
O11A—W7A—O28A—W12A	−60.0 (13)	O22B—W11B—O35B—W10B	−86.6 (8)
O29A—W8A—O30A—W9A	171.0 (7)	O25B—W11B—O35B—W10B	−6.9 (17)
O31A—W8A—O30A—W9A	−85.4 (7)	O39B—W11B—O35B—W10B	69.8 (8)
O27A—W8A—O30A—W9A	−5.7 (17)	O37B—W11B—O35B—W10B	−3.1 (6)
O13A—W8A—O30A—W9A	70.4 (7)	O34B—W10B—O35B—W11B	−171.9 (7)
O15A—W8A—O30A—W9A	−1.8 (6)	O33B—W10B—O35B—W11B	13.8 (17)
O32A—W9A—O30A—W8A	−170.7 (7)	O19B—W10B—O35B—W11B	86.4 (8)
O14A—W9A—O30A—W8A	−70.0 (8)	O36B—W10B—O35B—W11B	−70.0 (8)
O18A—W9A—O30A—W8A	10.3 (17)	O37B—W10B—O35B—W11B	3.1 (6)
O33A—W9A—O30A—W8A	86.5 (7)	O34B—W10B—O36B—W12B	173.5 (8)
O15A—W9A—O30A—W8A	1.8 (6)	O33B—W10B—O36B—W12B	−83.8 (8)
O29A—W8A—O31A—W12A	−126.3 (14)	O19B—W10B—O36B—W12B	−0.6 (18)
O30A—W8A—O31A—W12A	131.2 (14)	O35B—W10B—O36B—W12B	71.7 (8)
O27A—W8A—O31A—W12A	−25.0 (14)	O37B—W10B—O36B—W12B	−0.7 (7)
O13A—W8A—O31A—W12A	48 (2)	O40B—W12B—O36B—W10B	−170.9 (8)
O15A—W8A—O31A—W12A	58.1 (14)	O31B—W12B—O36B—W10B	84.2 (8)
O40A—W12A—O31A—W8A	127.8 (14)	O28B—W12B—O36B—W10B	8.5 (17)
O28A—W12A—O31A—W8A	24.6 (14)	O39B—W12B—O36B—W10B	−72.1 (8)
O39A—W12A—O31A—W8A	−54 (2)	O37B—W12B—O36B—W10B	0.7 (7)
O36A—W12A—O31A—W8A	−131.4 (14)	O15B—P1B—O37B—W10B	57.2 (9)
O37A—W12A—O31A—W8A	−59.0 (14)	O11B—P1B—O37B—W10B	177.9 (7)
O32A—W9A—O33A—W10A	130.1 (14)	O6B—P1B—O37B—W10B	−61.5 (10)
O14A—W9A—O33A—W10A	−51 (2)	O15B—P1B—O37B—W11B	178.9 (7)
O18A—W9A—O33A—W10A	27.9 (14)	O11B—P1B—O37B—W11B	−60.3 (9)
O30A—W9A—O33A—W10A	−129.1 (14)	O6B—P1B—O37B—W11B	60.3 (9)
O15A—W9A—O33A—W10A	−56.6 (14)	O15B—P1B—O37B—W12B	−61.9 (9)
O34A—W10A—O33A—W9A	−129.8 (14)	O11B—P1B—O37B—W12B	58.9 (9)
O35A—W10A—O33A—W9A	44 (2)	O6B—P1B—O37B—W12B	179.5 (7)
O36A—W10A—O33A—W9A	127.8 (14)	O34B—W10B—O37B—P1B	177 (4)
O19A—W10A—O33A—W9A	−27.1 (14)	O33B—W10B—O37B—P1B	−40.8 (9)
O37A—W10A—O33A—W9A	55.2 (14)	O19B—W10B—O37B—P1B	46.6 (8)
O34A—W10A—O35A—W11A	−170.2 (7)	O36B—W10B—O37B—P1B	−133.5 (9)
O36A—W10A—O35A—W11A	−68.0 (8)	O35B—W10B—O37B—P1B	134.7 (9)
O19A—W10A—O35A—W11A	86.4 (8)	O34B—W10B—O37B—W11B	40 (4)
O33A—W10A—O35A—W11A	16.5 (16)	O33B—W10B—O37B—W11B	−177.5 (5)
O37A—W10A—O35A—W11A	4.3 (7)	O19B—W10B—O37B—W11B	−90.1 (4)
O38A—W11A—O35A—W10A	169.6 (8)	O36B—W10B—O37B—W11B	89.9 (5)
O22A—W11A—O35A—W10A	−87.1 (8)	O35B—W10B—O37B—W11B	−2.0 (4)
O25A—W11A—O35A—W10A	−11.2 (17)	O34B—W10B—O37B—W12B	−49 (4)
O39A—W11A—O35A—W10A	68.5 (8)	O33B—W10B—O37B—W12B	93.1 (5)
O37A—W11A—O35A—W10A	−4.3 (7)	O19B—W10B—O37B—W12B	−179.5 (5)
O34A—W10A—O36A—W12A	170.7 (8)	O36B—W10B—O37B—W12B	0.5 (4)
O35A—W10A—O36A—W12A	67.7 (8)	O35B—W10B—O37B—W12B	−91.3 (5)
O19A—W10A—O36A—W12A	−12.4 (16)	O38B—W11B—O37B—P1B	−170 (3)
O33A—W10A—O36A—W12A	−86.6 (8)	O22B—W11B—O37B—P1B	−42.9 (8)
O37A—W10A—O36A—W12A	−3.6 (7)	O35B—W11B—O37B—P1B	−134.7 (9)
O40A—W12A—O36A—W10A	−169.1 (8)	O25B—W11B—O37B—P1B	43.7 (8)
O28A—W12A—O36A—W10A	13.9 (16)	O39B—W11B—O37B—P1B	132.6 (9)
O39A—W12A—O36A—W10A	−68.9 (8)	O38B—W11B—O37B—W10B	−34 (3)
O31A—W12A—O36A—W10A	86.8 (8)	O22B—W11B—O37B—W10B	93.8 (4)
O37A—W12A—O36A—W10A	3.6 (7)	O35B—W11B—O37B—W10B	2.0 (4)
O6A—P1A—O37A—W11A	60.7 (10)	O25B—W11B—O37B—W10B	−179.6 (4)
O11A—P1A—O37A—W11A	−58.9 (10)	O39B—W11B—O37B—W10B	−90.7 (5)
O15A—P1A—O37A—W11A	−179.9 (7)	O38B—W11B—O37B—W12B	56 (3)
O6A—P1A—O37A—W10A	−61.5 (9)	O22B—W11B—O37B—W12B	−177.0 (5)
O11A—P1A—O37A—W10A	178.9 (7)	O35B—W11B—O37B—W12B	91.2 (5)
O15A—P1A—O37A—W10A	57.9 (10)	O25B—W11B—O37B—W12B	−90.3 (4)
O6A—P1A—O37A—W12A	179.3 (7)	O39B—W11B—O37B—W12B	−1.5 (4)
O11A—P1A—O37A—W12A	59.7 (9)	O40B—W12B—O37B—P1B	−176 (3)
O15A—P1A—O37A—W12A	−61.3 (9)	O31B—W12B—O37B—P1B	45.2 (8)
O38A—W11A—O37A—P1A	−177 (3)	O28B—W12B—O37B—P1B	−42.1 (8)
O22A—W11A—O37A—P1A	−43.7 (9)	O36B—W12B—O37B—P1B	134.7 (9)
O25A—W11A—O37A—P1A	42.4 (9)	O39B—W12B—O37B—P1B	−133.7 (9)
O35A—W11A—O37A—P1A	−134.8 (10)	O40B—W12B—O37B—W10B	48 (3)
O39A—W11A—O37A—P1A	132.3 (10)	O31B—W12B—O37B—W10B	−90.0 (5)
O38A—W11A—O37A—W10A	−39 (3)	O28B—W12B—O37B—W10B	−177.3 (4)
O22A—W11A—O37A—W10A	93.8 (5)	O36B—W12B—O37B—W10B	−0.5 (4)
O25A—W11A—O37A—W10A	179.9 (5)	O39B—W12B—O37B—W10B	91.1 (5)
O35A—W11A—O37A—W10A	2.7 (4)	O40B—W12B—O37B—W11B	−41 (3)
O39A—W11A—O37A—W10A	−90.2 (4)	O31B—W12B—O37B—W11B	−179.6 (5)
O38A—W11A—O37A—W12A	50 (3)	O28B—W12B—O37B—W11B	93.1 (4)
O22A—W11A—O37A—W12A	−177.3 (5)	O36B—W12B—O37B—W11B	−90.1 (5)
O25A—W11A—O37A—W12A	−91.2 (5)	O39B—W12B—O37B—W11B	1.5 (4)
O35A—W11A—O37A—W12A	91.6 (5)	O38B—W11B—O39B—W12B	−170.1 (7)
O39A—W11A—O37A—W12A	−1.3 (4)	O22B—W11B—O39B—W12B	13.4 (17)
O34A—W10A—O37A—P1A	−180 (54)	O35B—W11B—O39B—W12B	−70.2 (8)
O35A—W10A—O37A—P1A	134.3 (9)	O25B—W11B—O39B—W12B	86.6 (7)
O36A—W10A—O37A—P1A	−131.7 (9)	O37B—W11B—O39B—W12B	2.3 (6)
O19A—W10A—O37A—P1A	44.4 (9)	O40B—W12B—O39B—W11B	170.2 (8)
O33A—W10A—O37A—P1A	−40.4 (8)	O31B—W12B—O39B—W11B	−4.9 (17)
O34A—W10A—O37A—W11A	43 (4)	O28B—W12B—O39B—W11B	−85.5 (7)
O35A—W10A—O37A—W11A	−2.7 (4)	O36B—W12B—O39B—W11B	70.6 (8)
O36A—W10A—O37A—W11A	91.3 (5)	O37B—W12B—O39B—W11B	−2.3 (6)
O19A—W10A—O37A—W11A	−92.6 (5)	C15S—N11S—C12S—O11S	−178.7 (16)
O33A—W10A—O37A—W11A	−177.4 (4)	C15S—N11S—C12S—N13S	2.3 (18)
O34A—W10A—O37A—W12A	−46 (4)	O11S—C12S—N13S—C14S	178.2 (17)
O35A—W10A—O37A—W12A	−91.7 (5)	N11S—C12S—N13S—C14S	−2.8 (19)
O36A—W10A—O37A—W12A	2.3 (4)	C12S—N13S—C14S—C15S	2.3 (19)
O19A—W10A—O37A—W12A	178.4 (5)	C12S—N11S—C15S—O12S	177.1 (16)
O33A—W10A—O37A—W12A	93.6 (4)	C12S—N11S—C15S—C14S	−0.9 (18)
O40A—W12A—O37A—P1A	−179 (2)	N13S—C14S—C15S—O12S	−178.8 (17)
O28A—W12A—O37A—P1A	−42.1 (8)	N13S—C14S—C15S—N11S	−0.8 (18)
O39A—W12A—O37A—P1A	−133.6 (9)	C25S—N21S—C22S—O21S	−178.9 (15)
O31A—W12A—O37A—P1A	44.1 (8)	C25S—N21S—C22S—N23S	3(2)
O36A—W12A—O37A—P1A	133.5 (9)	O21S—C22S—N23S—C24S	−179.3 (14)
O40A—W12A—O37A—W11A	−44 (3)	N21S—C22S—N23S—C24S	−0.6 (18)
O28A—W12A—O37A—W11A	92.8 (4)	C22S—N23S—C24S—C25S	−1.2 (17)
O39A—W12A—O37A—W11A	1.3 (4)	C22S—N21S—C25S—O22S	175.7 (16)
O31A—W12A—O37A—W11A	179.1 (5)	C22S—N21S—C25S—C24S	−3.1 (18)
O36A—W12A—O37A—W11A	−91.6 (5)	N23S—C24S—C25S—O22S	−176.2 (15)
O40A—W12A—O37A—W10A	46 (3)	N23S—C24S—C25S—N21S	2.6 (17)
O28A—W12A—O37A—W10A	−177.9 (4)	C35S—N31S—C32S—O31S	177.4 (15)
O39A—W12A—O37A—W10A	90.6 (4)	C35S—N31S—C32S—N33S	−2.8 (18)
O31A—W12A—O37A—W10A	−91.7 (4)	O31S—C32S—N33S—C34S	−178.4 (17)
O36A—W12A—O37A—W10A	−2.3 (4)	N31S—C32S—N33S—C34S	1.8 (19)
O40A—W12A—O39A—W11A	171.0 (7)	C32S—N33S—C34S—C35S	0(2)
O28A—W12A—O39A—W11A	−84.9 (7)	C32S—N31S—C35S—O32S	−177.4 (15)
O31A—W12A—O39A—W11A	−7.4 (16)	C32S—N31S—C35S—C34S	2.6 (19)
O36A—W12A—O39A—W11A	70.1 (7)	N33S—C34S—C35S—O32S	178.7 (15)
O37A—W12A—O39A—W11A	−2.1 (6)	N33S—C34S—C35S—N31S	−1.4 (19)
O38A—W11A—O39A—W12A	−170.9 (7)	C45S—N41S—C42S—O41S	178.4 (18)
O22A—W11A—O39A—W12A	11.5 (16)	C45S—N41S—C42S—N43S	−1(2)
O25A—W11A—O39A—W12A	86.5 (7)	O41S—C42S—N43S—C44S	−179.6 (18)
O35A—W11A—O39A—W12A	−69.7 (7)	N41S—C42S—N43S—C44S	0(2)
O37A—W11A—O39A—W12A	2.1 (6)	C42S—N43S—C44S—C45S	1(2)
O1B—W1B—O2B—W2B	129.0 (16)	C42S—N41S—C45S—O42S	−179.0 (19)
O3B—W1B—O2B—W2B	25.3 (16)	C42S—N41S—C45S—C44S	2(2)
O4B—W1B—O2B—W2B	−55 (2)	N43S—C44S—C45S—O42S	179.1 (19)
O5B—W1B—O2B—W2B	−129.2 (17)	N43S—C44S—C45S—N41S	−2(2)
O6B—W1B—O2B—W2B	−57.8 (16)	C55S—N51S—C52S—O51S	172.7 (17)
O7B—W2B—O2B—W1B	−126.0 (16)	C55S—N51S—C52S—N53S	−3.4 (19)
O9B—W2B—O2B—W1B	129.4 (17)	O51S—C52S—N53S—C54S	−171.4 (17)
O10B—W2B—O2B—W1B	46 (2)	N51S—C52S—N53S—C54S	5(2)
O8B—W2B—O2B—W1B	−25.3 (16)	O51S—C52S—N53S—C71S	0(3)
O11B—W2B—O2B—W1B	57.4 (16)	N51S—C52S—N53S—C71S	176.4 (15)
O1B—W1B—O3B—W3B	−126.0 (15)	C52S—N53S—C54S—C55S	−4.1 (19)
O2B—W1B—O3B—W3B	−22.1 (16)	C71S—N53S—C54S—C55S	−175.4 (15)
O4B—W1B—O3B—W3B	132.2 (16)	C52S—N51S—C55S—O52S	−177.9 (17)
O5B—W1B—O3B—W3B	53 (2)	C52S—N51S—C55S—C54S	0.8 (19)
O6B—W1B—O3B—W3B	60.5 (15)	N53S—C54S—C55S—O52S	−179.4 (17)
O12B—W3B—O3B—W1B	124.5 (16)	N53S—C54S—C55S—N51S	1.9 (18)
O8B—W3B—O3B—W1B	22.0 (16)	C65S—N61S—C62S—O61S	−177 (2)
O13B—W3B—O3B—W1B	−59 (2)	C65S—N61S—C62S—N63S	3(3)
O14B—W3B—O3B—W1B	−132.9 (16)	O61S—C62S—N63S—C64S	175 (2)
O15B—W3B—O3B—W1B	−61.2 (15)	N61S—C62S—N63S—C64S	−4(2)
O16B—W4B—O4B—W1B	−171.7 (8)	O61S—C62S—N63S—C71S	11 (4)
O19B—W4B—O4B—W1B	9.6 (18)	N61S—C62S—N63S—C71S	−168.5 (16)
O17B—W4B—O4B—W1B	−71.5 (8)	C62S—N63S—C64S—C65S	4(2)
O18B—W4B—O4B—W1B	85.4 (8)	C71S—N63S—C64S—C65S	167.9 (16)
O6B—W4B—O4B—W1B	1.7 (7)	C62S—N61S—C65S—O62S	178 (2)
O1B—W1B—O4B—W4B	171.3 (8)	C62S—N61S—C65S—C64S	0(2)
O3B—W1B—O4B—W4B	−83.9 (8)	N63S—C64S—C65S—O62S	179.9 (18)
O2B—W1B—O4B—W4B	−5.0 (17)	N63S—C64S—C65S—N61S	−2(2)
O5B—W1B—O4B—W4B	70.0 (8)	C62S—N63S—C71S—N53S	−116 (2)
O6B—W1B—O4B—W4B	−1.7 (7)	C64S—N63S—C71S—N53S	81 (2)
O20B—W5B—O5B—W1B	172.7 (7)	C52S—N53S—C71S—N63S	−79 (2)
O21B—W5B—O5B—W1B	−85.3 (8)	C54S—N53S—C71S—N63S	91 (2)
